# Exposure to Organophosphate Ester Flame Retardants and Plasticizers during Pregnancy and Autism-Related Outcomes in the ECHO Cohort

**DOI:** 10.1021/EHP.6c00247

**Published:** 2026-05-07

**Authors:** Jennifer L. Ames, Assiamira Ferrara, Juanran Feng, Stacey Alexeeff, Lyndsay A. Avalos, Emily S. Barrett, Theresa M. Bastain, Deborah H. Bennett, Jessie P. Buckley, Courtney C. Carignan, Patricia Cintora, Akhgar Ghassabian, Monique M. Hedderson, Ixel Hernandez-Castro, Kurunthachalam Kannan, Margaret R. Karagas, Catherine J. Karr, Jordan R. Kuiper, Donghai Liang, Kristen Lyall, Cindy T. McEvoy, Rachel Morello-Frosch, Thomas G. O’Connor, Jiwon Oh, Alicia K. Peterson, Lesliam Quiros-Alcala, Sheela Sathyanarayana, Susan Schantz, Rebecca J. Schmidt, Anne P. Starling, Tracey J. Woodruff, Heather E. Volk, Yeyi Zhu, Lisa A. Croen, ECHO Cohort Consortium

**Affiliations:** 1 Division of Research, 214681 Kaiser Permanente Northern California, Pleasanton, California 94612-5190, United States; 2 Department of Biostatistics and Epidemiology, Rutgers School of Public Health, and the Environmental and Occupational Health Sciences Institute, Rutgers University, Piscataway, New Jersey 08854, United States; 3 Department of Population and Public Health Sciences, 5116 University of Southern California, Los Angeles, California 90007, United States; 4 Department of Public Health Sciences, University of California, Davis, Davis, California 95616, United States; 5 Department of Epidemiology, 41474 University of North Carolina at Chapel Hill, Chapel Hill, North Carolina 27516, United States; 6 Department of Food Science and Human Nutrition, Department of Pharmacology and Toxicology, Michigan State University, East Lansing, Michigan 48824-1312, United States; 7 14589 University of Illinois Urbana−Champaign, Urbana, Illinois 61801, United States; 8 Departments of Pediatrics and Population Health, New York University Grossman School of Medicine, New York, New York 10016, United States; 9 Wadsworth Center, Division of Environmental Health Sciences, 1094 New York State Department of Health, Albany, New York 12237, United States; 10 Department of Environmental Health Sciences, University at Albany, State University of New York, Albany, New York 12222, United States; 11 Geisel School of Medicine at Dartmouth College, Hanover, New Hampshire 03755, United States; 12 Department of Pediatrics and Department of Environmental and Occupational Health Sciences, 7284 University of Washington, Seattle, Washington 98105, United States; 13 Department of Environmental and Occupational Health, Milken Institute School of Public Health, 50430 The George Washington University, Washington D.C. 20052, United States; 14 Gangarosa Department of Environmental Health, Rollins School of Public Health, 25798 Emory University, Atlanta, Georgia 30322-4201, United States; 15 A.J. Drexel Autism Institute, 6527 Drexel University, Philadelphia, Pennsylvania 19104, United States; 16 Department of Pediatrics, Pape Pediatric Research Institute, 6684 Oregon Health & Science University, Portland, Oregon 97239, United States; 17 School of Public Health and Department of Environmental Science, Policy and Management, 1438 University of California, Berkeley, California 94720-3114, United States; 18 Departments of Psychiatry, Neuroscience, and Obstetrics and Gynecology, 6927 University of Rochester, Rochester, New York 14627-0001, United States; 19 Department of Environmental Health and Engineering, 25802 Johns Hopkins Bloomberg School of Public Health, Johns Hopkins University, Baltimore, Maryland 70047, United States; 20 Department of Pediatrics, Epidemiology, Environmental and Occupational Health Sciences, and Center for Child Health, Behavior, and Development, Seattle Children’s Research Institute, 7284 University of Washington, Seattle, Washington 91807, United States; 21 The MIND Institute, 8789 University of California Davis, Sacramento, California 95817-2200, United States; 22 Department of Epidemiology, Gillings School of Global Public Health, 41474 University of North Carolina at Chapel Hill, Chapel Hill, North Carolina 27599-7400 United States; 23 Program on Reproductive Health and the Environment, Department of Obstetrics, Gynecology and Reproductive Sciences, University of California, San Francisco, California 94143, United States; 24 Department of Mental Health, 25802 Johns Hopkins Bloomberg School of Public Health, Johns Hopkins University, Baltimore, Maryland 70047, United States

## Abstract

**BACKGROUND**: Organophosphate ester flame retardants and plasticizers (OPEs) have myriad uses in industry and consumer products. Increasing human exposure to OPEs has raised concerns about their potential effects on child neurodevelopment during the pregnancy. **Objective:** We investigated whether OPE urinary concentrations during pregnancy were associated with child’s autism-related outcomes. **METHODS:** We included 4159 mother–child pairs from 15 cohorts in the NIH Environmental influences on Child Health Outcomes (ECHO) Consortium, with children born from 2006–2020 (median age [interquartile range]: 6 [4,10] years). Nine OPE biomarkers were measured in urine samples collected mid- to late pregnancy. Dilution-adjusted biomarkers were modeled continuously, categorically (high [>median], moderate [≤median], nondetect), or as detect/nondetect depending on their detection frequency. We assessed child autism-related traits via a) parent report on the Social Responsiveness Scale (SRS) and b) clinical autism diagnosis. We examined associations of OPEs with child outcomes, including modification by child sex, using generalized estimating equations to account for clustering by ECHO cohort. **RESULTS:** Compared with nondetectable concentrations, high exposure to bis­(butoxyethyl) phosphate (BBOEP) was associated with higher autistic trait scores (adj-β 0.97, 95% confidence interval [CI]: 0.42, 1.52) and greater odds of autism diagnosis (adjusted odds ratio [adj-OR]: 1.27, 95% CI: 1.07, 1.50). Bis­(1-chloro-2-propyl) phosphate (BCPP) showed associations with autistic trait scores (BCPP adj-β for high exposure vs nondetect: 0.34, 95% CI: −0.46, 1.13; BCPP adj-β for moderate exposure vs nondetect: 0.72, 95% CI: 0.24, 1.20). High exposure to bis­(2-chloroethyl) phosphate (BCETP) was associated with lower odds of autism diagnosis (adj-OR: 0.76, 95% CI: 0.60, 0.95). Other OPEs showed no associations in adjusted models. Associations between BBOEP and higher autistic trait scores were stronger in males than females. **DISCUSSION:** Prenatal exposure to OPEs, specifically BCPP and BBOEP, may be associated with a higher risk of autism diagnosis and related traits in childhood.

## Introduction

Organophosphate ester flame retardants and plasticizers (OPEs) are synthetic chemicals of emerging concern. Human exposure to OPEs has increased rapidly since these compounds replaced polybrominated diphenyl ether (PBDE) flame retardants, which were phased out in the early 2000s due to health risks.^1^ Today, the production and environmental concentrations of OPEs exceed those of PBDEs at their peak use.^1^ Similar to PBDEs, OPEs are not chemically bound to the many consumer products and textiles to which they are added; therefore, they gradually volatilize into indoor air and contaminate indoor dust. Human exposures mainly occur through ingestion of indoor dust and consumption of contaminated drinking water and food, although inhalation and dermal exposure are also important.^2,3^ Most Americans, including pregnant women, have detectable levels of OPEs in their bodies.^4–6^ OPEs are also present in placental tissues, suggesting that transfer to the fetus is possible.^7^ Although the biological half-life of OPEs (on the order of hours to days) is much shorter than the half-life of the PBDEs (years) they replaced, they appear to be similarly or even more toxic with continuous and ubiquitous exposure.^1,8–10^

In vitro and animal studies have demonstrated that exposure to OPEs has developmental, reproductive, and neurological effects.^11^ A limited number of epidemiological studies have found that early life OPE exposure is associated with adverse child development, including shorter gestational duration,^12^ greater risk of childhood obesity,^13,14^ and adverse neurodevelopment.^13^ Specifically, studies across the United States, Norway, and China have linked prenatal exposure to OPEs such as diphenyl phosphate (DPHP) and bis­(1,3-dichloro-2-propyl) phosphate (BDCPP) to multiple adverse neurodevelopmental outcomes, including decreases in cognitive and psychomotor functioning and greater problems with executive function, attention-deficit/hyperactivity disorder (ADHD)-related symptoms, and internalizing and externalizing behaviors.^15–18^

Toxicological and some human studies indicate that OPEs can alter sex steroid physiology; neuroendocrine signaling; pathways of inflammation and oxidative stress, including via induction of peroxisome proliferator-activated receptors (PPARs); and thyroid function.^11^ Interference with these critical pathways of fetal development is the same mechanism of action for other known developmental toxicants such as PBDEs, phthalates, and per- and polyfluoroalkyl substances (PFASs),^19^ suggesting that OPEs could similarly exert neurotoxic effects on the fetus during pregnancy. Further, evidence of sex-specific effects of OPEs, including potential antiandrogenic activity, has been noted in several human and animal studies.^20,21^ Some have found poorer neuropsychological functioning with OPE exposure in males relative to that in females.^16,22–24^ These potential sex differences warrant examination in larger samples.

Autism, a neurodevelopmental condition marked by lifelong disabilities in social communication, behavior, and sensory processing, is typically diagnosed in early childhood and affects 1 in 36 children (2.8%) in the United States today.^25^ While a small fraction of autism can be attributed to known genetic syndromes,^26^ most cases appear to arise from a complex combination of genetic and environmental influences in the prenatal period and early life.^27^ Autism is diagnosed more frequently in males than in females, which may point to endocrine disrupting pathways in the prenatal period.^28^ Only one prior study has examined the association between prenatal OPE exposure and autism, reporting null associations. However, the sample was small (*n* = 277) and had an elevated familial likelihood of autism.^29^

Building on this previous work, this study leverages a large, geographically diverse sample of U.S. children participating in 15 cohorts in the Environmental Influences on Child Health Outcomes (ECHO) Consortium to examine a broader range of urinary OPE metabolites during pregnancy and their association with child autism. We assess both quantitative measures of autism-related traits and clinical diagnoses while also examining potential effect modification by child sex.

## Methods

### Study Population

The NIH ECHO Cohort, comprising 69 cohorts, is a geographically diverse, national pediatric cohort established to investigate the effects of early life environmental factors on child health and development.^30,31^ Eligible participants included in this analysis had a) maternal OPE concentrations measured in a pregnancy urine sample and b) child data on either (i) autism-related traits ascertained anytime between the ages of 2.5–18 years using the Social Responsiveness Scale (SRS) parent reports or (ii) an autism diagnosis made by a health care provider, as indicated by parent report or the child’s medical records. The study population was restricted to singleton births. We further restricted the population to participants from ECHO cohorts with at least 15 mother–child pairs contributing to the analytic sample (resulting in the exclusion of one cohort). In total, 4159 mother–child pairs from 15 cohorts across 12 states were included in this analysis. A description of the study cohorts is included in Table S1 and a flowchart of study exclusions in Figure S1.

All study procedures were approved by either the cohort’s local institutional review board (IRB) or ECHO’s single IRB prior to the start of the study. Each ECHO cohort obtained written informed consent from the participating parent or caregiver and assent from the participating child.

### Assessment of OPE Exposure

Each of the included 15 cohorts had selected, using their own criteria, a subsample of their participants for ECHO’s OPE biomarker assessment (details previously described in Oh et al.).^12^ OPEs were quantified in a single spot or first morning urine sample collected from the participant in predominantly the second or third trimester of pregnancy (mean [standard deviation (SD)] = 27.3 [4.9] gestational weeks). These samples were shipped on dry ice to the Wadsworth Center’s Human Health Exposure Analysis Resource (HHEAR) laboratory, where they were analyzed for nine OPE metabolites. These laboratory methods have been previously described.^12^ Briefly, the lab performed solid-phase extraction followed by identification and quantification of target compounds in urine samples using high-performance liquid chromatography (HPLC; ExionLC system, SCIEX), combined with an AB SCIEX TRAP 5500+triple quadrupole mass spectrometer (Applied Biosystems).^24^ Nine OPE biomarkers and nine internal standards were separated using a Kinetex hydrophilic interaction liquid chromatography column (100 mm × 2.1 mm, μ2.6 μm particle size; Phenomenex) coupled with a Betasil C18 guard column (20 mm × 2.1 mm, μ5 μm particle size; Thermo Scientific). Quality control (QC) samples including standard reference material were analyzed with every batch. Trace levels of select OPEs were found in reagent blanks, and the reported concentrations in samples were corrected by subtracting blank values by batch. Seven cohorts also provided masked duplicate samples (*n* = 191) used to assess the precision of the OPE assessment. Further QA/QC details—including average recoveries, coefficients of variation, and relative percentage differences among the duplicate samples—are presented in Oh et al. (2024).^12^

The nine OPE biomarkers assayed include a) bis­(butoxyethyl) phosphate (BBOEP), a metabolite of tris­(2-butoxyethyl) phosphate (TBOEP); b) bis­(2-chloroethyl) phosphate (BCETP), a metabolite of tris­(2-chloroethyl) phosphate (TCETP); c) bis­(1-chloro-2-propyl) phosphate (BCPP), a metabolite of tris­(1-chloro-2-propyl) phosphate (TCPP); d) bis­(1,3-dichloro-2-propyl) phosphate (BDCPP), a metabolite of tris­(1,3-dichloro-2-propyl) phosphate (TDCPP); e) bis­(2-ethylhexyl) phosphate (BEHP), a metabolite of tris­(2-ethylhexyl) phosphate (TEHP); f) bis­(2-methylphenyl) phosphate (BMPP), a metabolite of tris­(2-methylphenyl) phosphate (TMPP); g) a composite of dibutyl phosphate and diisobutyl phosphate (DBUP/DIBP), metabolites of tributyl phosphate (TBUP) and its isomer tri-isobutyl phosphate (TIBP); h) di­(2-propylheptyl) phthalate (DPHP), a major metabolite of triphenyl phosphate (TPHP); and (i) dipropyl phosphate (DPRP), a metabolite of tripropyl phosphate (TPRP). DBUP/DIBP is reported as a composite sum of both analytes because the analytes coeluted and could not be quantified individually. The limits of detection (LODs) ranged from 0.01 to 0.04 ng/mL across analytes. Relative percent differences were generally acceptable among duplicate samples but exceeded 30% for two analytes—BEHP (53.6%) and DPRP (32.7%)—both of which had most sample values measure close to or below the LOD,^12^ potentially reducing precision.^32^

We corrected OPE biomarker concentrations for urinary dilution using either specific gravity (*n* = 3682 from 12 cohorts) or creatinine (*n* = 477 from 4 cohorts), depending on what was measured by the individual ECHO cohorts. To harmonize across these methods, we used an approach validated by Kuiper et al.^33–35^ Briefly, for cohorts with creatinine measurements, we multiplied the OPE concentrations by the ratio of the cohort-specific median creatinine value to the participant’s creatinine value to calculate the dilution-adjusted concentration. For cohorts with specific gravity measurements, we followed the same procedure but first subtracted one from the cohort-specific median specific gravity value and the participant’s specific gravity value.

### Autism-Related Outcomes

We assessed children’s social and communication skills as a quantitative trait with the SRS.^36^ The SRS is a 65-item Likert response instrument completed by parents about their child’s social use of language, reciprocal social behavior, and restricted interests or repetitive behaviors. Higher scores on the SRS correspond to higher levels of autism-related traits. The SRS shows psychometric reliability and validity in identifying clinically significant social impairments in both general population and clinical samples of children.^38–40^ ECHO cohorts administered either the preschool (aged 2.5–4.5 years) or school-age (aged 4–18 years) version of the SRS depending on the age of the participant. If a participant had multiple SRS assessments, we used the most recent administration. An individual’s SRS score tends to remain stable over time, as demonstrated with repeat administrations of the school-age form at different ages.^41–43^ Scores on the preschool and school-age versions also show high correlation (*r* = 0.60) in ECHO participants.^44^ A validated, 16-item short form of the School-Age SRS (Western Psychological Services)^37^ was completed by 586 participants.

SRS raw scores are converted to clinically informative T-scores, which are standardized by sex for ages 4–18 years and by SRS version (preschool vs school-age) using an independent normative sample (mean of 50, with an SD of 10).^36^ We also examined clinically meaningful cutoffs in the distribution of the SRS T-score indicative of mild (T-Score = 60–65) and moderate/severe autistic traits (T-score ≥ 66), as defined by the SRS publisher, as well as the Social Communication and Interaction and Restricted Interests and Repetitive Behaviors subscales.

As a second primary outcome, we assessed the clinical diagnosis of autism, obtained either by parent-reported medical history or from abstraction of child medical records.

### Covariates

Using a directed acyclic graph, we identified potential confounders and precision variables for our analysis (Figure S2). These included maternal age at delivery (in years), maternal educational attainment (up to high school degree/general educational development, some college/associated degree/trade school, bachelor’s degree, and master’s/professional/doctoral degree), maternal prepregnancy body mass index (BMI) (categorized as <18.5, 18.5–24.9, 25.0–29.9, and ≥30.0 kg/m^2^), maternal smoking during pregnancy (yes, no), parity (0, ≥1), child’s sex (female, male), and child’s age at assessment. Covariate data were obtained from participant questionnaires and abstracted from medical records (see Table S2 for details). We additionally included self-reported maternal race/ethnicity, recognizing it as a social construct associated with experiences of structural inequities, racism, and environmental injustice. Categories included Hispanic, non-Hispanic Black, non-Hispanic Asian, non-Hispanic White, and non-Hispanic Other races (Pacific Islander, American Indian, multiple-races, and other/not reported race) which were collapsed into one category due to small numbers. Race/ethnicity has been linked to both differences in exposure to endocrine-disrupting chemicals and disparities in SRS sensitivity and autism diagnosis.^45,46^

### Statistical Analyses

We first examined descriptive characteristics and the distribution of the dilution-standardized OPE biomarkers within the pooled ECHO sample, as well as within each individual ECHO cohort. We further examined the Spearman correlation coefficients among the OPE biomarkers.

We modeled the association between each OPE biomarker and autism-related outcome using generalized estimating equations to account for the clustering of individuals by ECHO cohort. Models were fitted with exchangeable correlation matrices and the identity link for continuous outcomes or the logit link for binary outcomes. Robust standard errors were used to estimate 95% confidence intervals (CIs). Given the varying detectability of OPE metabolites, we modeled each OPE following an approach used in Oh et al. (2024).^12^ For OPE metabolites with >80% detection above the LOD, we assigned values below the LOD with the machine-read values provided by the laboratory and then transformed all values by log 2. If a machine-read value was negative or zero, we replaced it with 0.001 prior to transformation. We separately modeled these metabolites continuously and as tertiles to examine linear and nonlinear relationships, respectively. For OPE biomarkers detected in 50–80% of participants, we created three-level categorical variables, with the nondetect category defined as participants with values <LOD and the remaining two categories created by dichotomizing participants at the median of the detectable dilution-adjusted values (high- and moderate-exposure categories). Lastly, for OPE biomarkers detected in <50% of participants, we dichotomized these as binary variables of detect (>LOD) vs nondetect. For models of the three-category OPE variables, we additionally reported the *p*-values from a Type III Wald test of the overall association for each OPE variable.

We examined sex differences in models stratified by child sex and additionally in interaction models including a product term between the child sex and OPE metabolite.

Fully adjusted models included the covariates mentioned above. Models for the SRS T-score were not adjusted for child sex, because the T-score is normed by sex for the school-age form, which was completed by 71% of our sample. For any covariates with missing data (all variables had less than 20% missing observations), we imputed missing values with multiple imputation by chained equations (MICE)^47^ using all the covariate data, including ECHO cohort, as predictors and implementing 50 imputed data sets and 100 burn-in iterations.

In the secondary analyses of the SRS outcome, we further examined the robustness and specificity of the associations between OPEs and 1) SRS raw scores, 2) T-scores on the two subscales of the SRS (Social Communication and Interaction and Restricted Interests and Repetitive Behaviors), and 3) SRS cutoff points for mild and moderate/severe autistic traits.

In sensitivity analyses, we stratified the results by SRS version completed (preschool vs school-age) to account for potential differences in the sensitivity of the SRS at younger ages. For both the SRS T-score and autism diagnosis models, we reran analyses excluding two ECHO cohorts enrolling pregnancies of younger siblings of children with autism, as these participants had an elevated familial likelihood of autism—the Markers of Autism Risk in Babies: Learning Early Signs (MARBLES) and Early Autism Risk Longitudinal Investigation (EARLI) studies. We performed leave-one-cohort-out analyses to assess the stability of the results when each cohort was excluded one at a time from the adjusted model. Lastly, for the autism diagnosis model, we conducted an additional sensitivity analysis restricted to cohorts contributing ≥15 autism cases.

We used SAS (version EG 8.3; SAS Institute, Inc.) to conduct all analyses and R (version 4.4.0; R Development Core Team) to create all plots.

## Results

Table 1 presents the descriptive characteristics of the overall sample as well as the subsamples contributing to the analyses of SRS assessment (*n* = 3288 mother–child pairs) and autism diagnosis (*n* = 3680 pairs). There were 2089 participants who contributed to both analyses. The overall sample was racially/ethnically diverse, with 13% self-identifying as Hispanic, 22% as non-Hispanic Black, 5% as non-Hispanic Asian, 56% as non-Hispanic White, and 4% as non-Hispanic Pacific Islander, American Indian, multiracial, or other. Most mothers were 30 years or older when they gave birth (59%), were multiparous (56%), had at least a bachelor’s degree (53%), did not smoke during pregnancy (86%), and gave birth in 2011–2015 (46%). Among their children (median age [interquartile range]: 6 [4,10] years), the average age at SRS assessment was 7 years (SD: 3.3, range 2–16), with a mean T-score (SD) of 48.8 (8.6) in all 15 cohorts and 48.6 (8.3) among the 13 population-based cohorts, excluding the two cohorts with increased familial likelihood of autism (MARBLES and EARLI). The prevalence of autism was 4.9% (*n* = 179) among participants from all 15 cohorts with autism diagnostic information and 2.3% (*n* = 86) among the 13 population-based cohorts, excluding MARBLES and EARLI. Descriptive characteristics across the 15 ECHO cohorts are presented in Table S3. We observed good agreement between autism diagnosis and SRS T-scores among the overlap of the two outcome samples, with median (IQR) T-scores of 64 (53–72) in participants with autism (*n* = 120) and 47 (43–53) in participants without autism (*n* = 2689) (Table S4).

**Table 1:** Descriptive Characteristics of the Pooled Cohort Overall and Participants Contributing to SRS and Autism Analyses, NIH ECHO^a^

	**Overall**	**SRS Sample**	**Autism Sample**
	(*n* = 4159)	(*n* = 3288)	(*n* = 3680)
**Maternal Age (years)**			
16–24	670 (16.1%)	461 (14.0%)	639 (17.4%)
25–29	1040 (25.0%)	815 (24.8%)	929 (25.2%)
30–34	1401 (33.7%)	1157 (35.2%)	1218 (33.1%)
35–47	1048 (25.2%)	855 (26.0%)	894 (24.3%)
**Maternal Education**			
Up to high school degree, GED, or equivalent	1130 (27.2%)	777 (23.6%)	1073 (29.2%)
Some college, no degree	690 (16.6%)	526 (16.0%)	624 (17.0%)
Bachelor’s degree	1155 (27.8%)	953 (29.0%)	1001 (27.2%)
Master’s and above	1034 (24.9%)	900 (27.4%)	892 (24.2%)
Missing	150 (3.6%)	132 (4.0%)	90 (2.4%)
**Maternal Race/Ethnicity**			
Hispanic All	543 (13.1%)	391 (11.9%)	510 (13.9%)
Non-Hispanic Black	899 (21.6%)	596 (18.1%)	883 (24.0%)
Non-Hispanic Asian	213 (5.1%)	157 (4.8%)	208 (5.7%)
Non-Hispanic Other (Pacific Islander, American Indian, multirace and other)	155 (3.7%)	119 (3.6%)	146 (4.0%)
Non-Hispanic White	2328 (56.0%)	2006 (61.0%)	1918 (52.1%)
Missing/Unknown	21 (0.5%)	19 (0.6%)	15 (0.4%)
**Parity**			
Nulliparous	1743 (41.9%)	1415 (43.0%)	1528 (41.5%)
Multiparous	2321 (55.8%)	1803 (54.8%)	2074 (56.4%)
Missing	95 (2.3%)	70 (2.1%)	78 (2.1%)
**Prepregnancy BMI** (kg/m^2^)			
<18.5	123 (3.0%)	91 (2.8%)	114 (3.1%)
18.5–24.9	1869 (44.9%)	1500 (45.6%)	1612 (43.8%)
25.0–29.9	1015 (24.4%)	810 (24.6%)	905 (24.6%)
≥30.0	1057 (25.4%)	817 (24.8%)	966 (26.3%)
Missing	95 (2.3%)	70 (2.1%)	83 (2.3%)
**Tobacco Use during Pregnancy**			
No	3575 (86.0%)	2841 (86.4%)	3173 (86.2%)
Yes	320 (7.7%)	253 (7.7%)	300 (8.2%)
Missing	264 (6.3%)	194 (5.9%)	207 (5.6%)
**Timing of Urine Collection**			
First Trimester	5 (0.1%)	<5	<5
second trimester	1755 (42.2%)	1439 (43.8%)	142 (39.7%)
Third trimester	2399 (57.7%)	<1850 (<57%)	<2220 (<61%)
**Urinary Dilution**			
Specific Gravity	3682 (88.5%)	2862 (87.0%)	3209 (87.2%)
Creatinine	477 (11.5%)	426 (13.0%)	471 (12.8%)
**Child Sex**			
Female	2065 (49.7%)	1633 (49.7%)	1825 (49.6%)
Male	2094 (50.3%)	1655 (50.3%)	1855 (50.4%)
**Year of Birth**			
2006–2010	1429 (34.4%)	952 (29.0%)	1399 (38.0%)
2011–2015	1900 (45.7%)	1697 (51.6%)	1565 (42.5%)
2016–2020	830 (20.0%)	639 (19.4%)	716 (19.5%)
**Total SRS Raw Score**			
Mean (SD)	31.3 ± 20.6	31.3 ± 20.6	NA
Median (Q1–Q3)	27.0 (17.0–40.0)	27.0 (17.0–40.0)	
Min-Max	0.0–156.0	0.0–156.0	
Missing	871	NA	
**Total SRS T-Score**			
Mean (SD)	48.8 ± 8.6	48.8 ± 8.6	NA
Median (Q1–Q3)	47.0 (43.0–53.0)	47.0 (43.0–53.0)	
Min-Max	34.0–99.0	34.0–99.0	
Missing	871	NA	
**SRS Social Communication and Interaction Subscale T-Score**			
Mean (SD)	48.1 ± 8.5	48.1 ± 8.5	
Median (Q1–Q3)	46.0 (42.0–51.0)	46.0 (42.0–51.0)	NA
Min–Max	35.0–100.0	35.0–100.0	
Missing	1411	540	
**SRS Restricted Interests and Repetitive Behaviors Subscale T-Score**			
Mean (SD)	48.7 ± 8.5	48.7 ± 8.5	
Median (Q1–Q3)	46.0 (43.0–52.0)	46.0 (43.0–52.0)	NA
Min–Max	40.0–104.0	40.0–104.0	
Missing	1411	540	
**SRS Second Edition (SRS-2) Version**			
SRS-2 Preschool (ages 2.5–4.5 y)	961 (23.1%)	961 (29.2%)	NA
SRS-2 School-Age (ages 4–18 y)	2327 (56.0%)	2327 (70.8%)	
Missing	871 (20.9%)	NA	
**SRS form**			
Full	2748 (66.1%)	2748 (83.6%)	NA
Short	540 (13.0%)	540 (16.4%)	
**Child’s Age at SRS (years)**			
Mean (SD)	6.9 ± 3.3	6.9 ± 3.3	
Median (Q1–Q3)	6.0 (4.0–10.0)	6.0 (4.0–10.0)	NA
Min, Max	2.9, 16	2.9, 16	NA
Missing	871	0	
**Clinical ASD Diagnosis**			
No	3501 (84.2%)	2689 (81.8%)	3501 (95.1%)
Yes	179 (4.3%)	120 (3.6%)	179 (4.9%)
Missing	479 (11.5%)	479 (14.6%)	NA
**Child’s Age at Last Medical History Followup**			6.8 (2.8)
Mean (SD)			6.2 (4.6–9.4)
Median (Q1–Q3)	NA	NA	1.1–13.0
Missing			528 (14.3%)

a Abbreviations: ASD, autism spectrum disorder; BMI, body mass index; ECHO, environmental influences on child health outcomes; GED, general educational development; NIH, National Institutes of Health; SD, standard deviation; SRS, Social Responsiveness Scale.

Demographic characteristics of children from the 15 participating ECHO cohorts who did not meet the inclusion criteria (*n* = 11,001) are presented in Table S5. Approximately 15% of participants who were not included were born and enrolled in ECHO in 2021–2023, after the OPE assay process had begun. While included participants were more likely than not-included participants to be Hispanic and to have missing data on key covariates, distributions of autism-related outcomes among those with nonmissing outcome data were very similar. On average, SRS T-scores were slightly lower in included vs not-included participants (48.1 ± 8.5 vs 49.0 ± 8.3), while autism diagnosis was slightly more prevalent (4.9% vs 4.5%).

The three OPE metabolites with highest detection were DBUP/DIBP (96%), DPHP (99.5%), and BDCPP (87%). BCPP, BCETP, and BBOEP were detected in 52–71% of samples. BMPP, BEHP, and DPRP were detected in 26–36% of samples (Table S6). The metabolites with the highest median concentrations were DPHP (0.95 μg/L), BDCPP (0.88 μg/L), and BCETP (0.56 μg/L) (Table S6 and Figure S3). There was some variability in OPE concentrations by ECHO cohort, particularly for OPEs with detection frequencies less than 70% (Figure S4). The metabolites were weakly correlated with each other (Spearman *R* = −0.07 to 0.25) (Figure S5).

### Associations of OPEs with SRS Scores

When modeled continuously, DBUP/DIBP, DPHP, and BDCPP exposures were not associated with SRS T-scores in the crude or adjusted models (Table 2). When modeled as tertiles, the second tertiles of DPHP and BDCPP exposure were associated with higher SRS T-scores (i.e., greater autistic traits) compared to the first tertiles in the crude models (β-adj [95% CI]: 0.59 [0.11, 1.06] for DPHP and 0.74 [0.28, 1.20] for BDCPP). However, these associations were attenuated and no longer significant in the fully adjusted models (Table 2). DBUP/DIBP exposure modeled as tertiles was not associated with the SRS T-score in the crude or adjusted models. Compared to nondetectable BCPP, moderate exposure to BCPP was associated with higher SRS scores (β-adj [95% CI]: 0.72 [0.24, 1.20]), and high exposure had a suggestive association (β-adj [95% CI]: 0.34 [-0.46,1.13]) (Table 2). BBOEP exposure showed a dose–response relationship with SRS T-scores, with moderate and high exposure corresponding with a 0.47 (95% CI: −0.62, 1.56) and 0.97 (95% CI: 0.42, 1.52) increase in SRS score in the adjusted models, respectively. The other OPEs were not associated with SRS T-scores.

**Table 2:** Associations of OPEs and SRS T-Scores in Linear Models Fitted with Generalized Estimating Equations with Clustering by ECHO Cohort^f^

			**SRS Total T-Score**		
**OPE Analyte**	**Level**	**Exposure Range** ^a^	**β**_**crude**_ **(95% CI)** ^b^ *n* = 3288	**β**_**adj**_ **(95% CI)** ^c^ *n* = 3288	***p*-Value** ^d^
**Continuous/Tertiles**					
DBUP/DIBP	Continuous		0.02 (−0.25, 0.28)	0.04 (−0.22, 0.29)	
	Tertile 3	0.25–16.3	–0.33 (−0.91, 0.25)	–0.16 (−0.84, 0.52)	0.64
	Tertile 2	0.15–0.25	0.05 (−0.39, 0.49)	0.11 (−0.32, 0.55)	
	Tertile 1	0.00–0.15	ref	ref	
DPHP	Continuous		0.24 (−0.09, 0.57)	–0.00 (−0.10, 0.10)	
	Tertile 3	1.42–1,661.7	0.71 (−0.23, 1.65)	–0.00 (−0.52, 0.52)	0.28
	Tertile 2	0.65–1.42	0.59 (0.11, 1.06)	0.27 (−0.16, 0.70)	
	Tertile 1	0.00–0.65	ref	ref	
BDCPP	Continuous		0.04 (−0.04, 0.11)	0.02 (−0.04, 0.09)	
	Tertile 3	1.31–44.76	0.56 (−0.01, 1.14)	0.12 (−0.42, 0.67)	0.15
	Tertile 2	0.47–1.31	0.74 (0.28, 1.20)	0.45 (−0.01, 0.91)	
	Tertile 1	0.00–0.47	ref	ref	
**Categorical**					
BCPP	>Median^e^	0.70–54.39	–0.10 (−1.09, 0.89)	0.34 (−0.46, 1.13)	0.012
	≤Median	0.02–0.70	0.34 (−0.35, 1.02)	0.72 (0.24, 1.20)	
	<LOD	<LOD	ref	ref	
BCETP	>Median^e^	1.04–207.0	0.44 (−0.18, 1.06)	0.08 (−0.42, 0.58)	0.84
	≤Median	0.03–1.04	0.04 (−0.59, 0.68)	0.14 (−0.43, 0.70)	
	<LOD	<LOD	ref	ref	
BBOEP	>Median^e^	0.07–14.89	1.48 (0.83, 2.13)	0.97 (0.42, 1.52)	<0.001
	≤Median	0.01–0.07	0.99 (−0.27, 2.24)	0.47 (−0.62, 1.56)	
	<LOD	<LOD	ref	ref	
**Detect/Nondetect**					
BMPP	≥LOD		0.38 (−0.90, 1.66)	0.03 (−0.73, 0.79)	
	<LOD		ref	ref	
BEHP	≥LOD		0.12 (−0.61, 0.85)	0.12 (−0.42, 0.66)	
	<LOD		ref	ref	
DPRP	≥LOD		–0.48 (−1.23, 0.27)	–0.22 (−0.83, 0.38)	
	<LOD		ref	ref	

a Abbreviations: BBOEP, bis­(butoxyethyl) phosphate; BCPP, bis­(1-chloro-2-propyl) phosphate; BCETP, bis­(2-chloroethyl) phosphate; BDCPP, bis­(1,3-dichloro-2-propyl) phosphate; BEHP, bis­(2-ethylhexyl) phosphate; BMPP, bis­(2-methylphenyl) phosphate; BMI, body mass index; CI, confidence interval; DBUP/DIBP, composite of dibutyl phosphate and diisobutyl phosphate; DPHP, di­(2-propylheptyl) phthalate; DPRP, dipropyl phosphate; ECHO, environmental influences on child health outcomes; LOD, limit of detection; MICE, multiple imputation by chained equations; OPE, organophosphate ester flame retardant and plasticizer; SRS, Social Responsiveness Scale.

b Dilution corrected, untransformed values, μg/L.

c Adjusted for the cohort.

d Adjusted MICE models included ECHO cohort, maternal age (continuous), child age at SRS assessment (continuous), race/ethnicity, prepregnancy BMI, educational attainment, sex, parity, and smoking during pregnancy. T-score is standardized for SRS version (preschool, school-age) and child’s sex for children 4 years+.

e Overall *p*-value of the three-category OPE exposure variable calculated from a type III Wald test.

f Median of detectable values.

The association between BBOEP exposure and SRS T-scores differed by child sex, with stronger associations among male children (β-adj [95% CI]: 1.58 [0.83, 2.33]) than female children (β-adj [95% CI]: 0.36 [−0.31, 1.04]) (*p*_int_ = 0.001) (Table 3). BDCPP exposure and moderate BCPP exposure were associated with higher SRS scores in males (β-adj [95% CI]: 0.09 [0.02, 0.15] for BDCPP and 0.91 [0.13, 1.69] for BCPP) but not females (β-adj [95% CI]: −0.03 [−0.15, 0.08] for BDCPP and 0.59 [−0.23, 1.41] for BCPP). However, the interaction terms between these metabolites and child sex were not statistically significant (*p*_int_ = 0.16 and 0.44, respectively).

**Table 3:** Associations of OPEs and SRS T-Scores in Linear Models Fitted with Generalized Estimating Equations with Clustering by ECHO Cohort, Stratified by Child Sex^e^

		**SRS Total T-Score**			
		**Male**	**Female**		
**OPE Analyte**	**Level**	**β**_**adj**_ **(95% CI)** ^a^ *n* = 1655	**β**_**adj**_ **(95% CI)** ^a^ *n* = 1633	***P*_interaction_** ^b^	***P*_interaction_** ^c^
**Continuous**					
DBUP/DIBP	Continuous	0.03 (−0.37, 0.44)	0.03 (−0.31, 0.38)	0.77	
DPHP	Continuous	–0.02 (−0.17, 0.12)	0.02 (−0.08, 0.11)	0.1	
BDCPP	Continuous	0.09 (0.02, 0.15)	–0.03 (−0.15, 0.08)	0.16	
**Categorical**					
BCPP	>Median^d^	0.63 (−0.06, 1.31)	0.12 (−1.11, 1.35)	0.18	0.27
	≤Median	0.91 (0.13, 1.69)	0.59 (−0.23, 1.41)	0.44	
	<LOD				
BCETP	>Median^d^	0.15 (−0.64, 0.94)	0.01 (−0.69, 0.71)	0.71	0.73
	≤Median	–0.11 (−0.78, 0.56)	0.42 (−0.65, 1.49)	0.44	
	<LOD				
BBOEP	>Median^d^	1.58 (0.83, 2.33)	0.36 (−0.31, 1.04)	0.0013	0.0016
	≤Median	0.70 (−0.57, 1.97)	0.24 (−0.89, 1.37)	0.22	
	<LOD				
**Detect/Nondetect**					
BMPP	≥LOD	0.43 (−0.43, 1.29)	–0.33 (−1.27, 0.60)	0.17	
	<LOD				
BEHP	≥LOD	0.67 (−0.20, 1.53)	–0.37 (−1.09, 0.34)	0.11	
	<LOD				
DPRP	≥LOD	–0.13 (−0.88, 0.62)	–0.29 (−0.86, 0.27)	0.37	
	<LOD				

a Abbreviations: BBOEP, bis­(butoxyethyl) phosphate; BCPP, bis­(1-chloro-2-propyl) phosphate; BCETP, bis­(2-chloroethyl) phosphate; BDCPP, bis­(1,3-dichloro-2-propyl) phosphate; BEHP, bis­(2-ethylhexyl) phosphate; BMPP, bis­(2-methylphenyl) phosphate; BMI, body mass index; CI, confidence interval; DBUP/DIBP, composite of dibutyl phosphate and diisobutyl phosphate; DPHP, di­(2-propylheptyl) phthalate; DPRP, dipropyl phosphate; ECHO, environmental influences on child health outcomes; LOD, limit of detection; MICE, multiple imputation by chained equations; OPE, organophosphate ester flame retardant and plasticizer; SRS, Social Responsiveness Scale.

b Adjusted MICE models included ECHO cohort, maternal age (continuous), child age at SRS assessment (continuous), race/ethnicity, prepregnancy BMI, educational attainment, parity, and smoking during pregnancy. T-score is standardized for SRS version (preschool, school-age) and child’s sex for children 4y+.

c *P*-value obtained from an interaction model that included both a first-order term for sex and the cross-product term of child sex and OPE analyte added to adjusted model. We considered *P*-interaction <0.05 to be statistically significant.

d Overall *P*-value of three-category OPE × sex interaction model using Type III Wald test. We considered *P*-interaction <0.05 to be statistically significant.

e Median of the detectable values.

In secondary analyses, compared with the nondetect category, the moderate-BCPP exposure category and the high-BBOEP exposure category remained associated with greater autistic traits in models of the SRS raw scores and with both SRS subscales (Social Communication and Interaction and Restricted Interests and Repetitive Behaviors) (Tables S7 and S8). Detectable levels of BEHP, compared to nondetectable levels, were modestly associated with higher scores on the Restricted Interests and Repetitive Behaviors subscale (β-adj [95% CI]: 0.53 [0.04, 1.03]). In analyses dichotomizing the SRS scores by clinically relevant cutoff points, compared with the nondetect category, the moderate-BCPP exposure category and both the high- and moderate-BBOEP exposure categories were also associated with greater odds of moderate/severe autistic traits but not mild traits. The high BCETP exposure category, however, was associated with greater odds of mild autistic traits (OR-adj [95% CI]: 1.43 [1.07, 1.91]) (Table S9).

### Associations of OPEs with Autism Diagnosis

When modeled continuously and as tertiles, DBUP/DIBP, DPHP, and BDCPP exposures were not associated with the autism diagnosis (Table 4). As compared with the nondetect category, the high-BBOEP exposure category was associated with higher odds of autism diagnosis (OR-adj [95% CI]: 1.27 [1.07, 1.50]), whereas the moderate-exposure category was not (OR-adj [95% CI]: 0.95 [0.75, 1.19]) (Table 4). In contrast, compared with the nondetect category, the high-BCETP exposure category was associated with lower odds of autism diagnosis (OR-adj [95% CI]: 0.76 [0.60, 0.95]), whereas the estimate for the moderate-exposure category was attenuated and not significant (OR-adj [95% CI]: 0.83 [0.64, 1.08]). The other OPEs were not associated with the odds of autism diagnosis.

**Table 4:** Associations of OPEs and Autism Diagnosis in Logistic Models Fitted with Generalized Estimating Equations with Clustering by ECHO Cohort^e^

			**Clinical Autism Diagnosis**		
**OPE Analyte**	**Level**	**Exposure Range** ^a^	**OR**_**crude**_ **(95% CI)** ^b^ *n* = 3,680	**OR**_**adj**_ **(95% CI)** ^c^ *n* = 3,680	***p*-Value** ^d^
**Continuous/Tertiles**					
DBUP/DIBP	Continuous		1.02 (0.96, 1.09)	1.02 (0.95, 1.10)	
	Tertile 3	0.26–16.3	0.96 (0.79, 1.15)	0.93 (0.73, 1.17)	0.59
	Tertile 2	0.15–0.26	1.12 (0.81, 1.57)	1.08 (0.76, 1.54)	
	Tertile 1	<LOD–0.15	ref	ref	
DPHP	Continuous		0.95 (0.87, 1.03)	0.93 (0.84, 1.02)	
	Tertile 3	1.47–1,661.7	0.83 (0.53, 1.29)	0.78 (0.48, 1.26)	0.29
	Tertile 2	0.70–1.47	0.98 (0.77, 1.26)	0.95 (0.71, 1.27)	
	Tertile 1	<LOD–0.70	ref	ref	
BDCPP	Continuous		1.01 (0.99, 1.02)	1.00 (0.99, 1.02)	
	Tertile 3	1.42–53.76	0.99 (0.84, 1.16)	0.94 (0.79, 1.12)	0.44
	Tertile 2	0.52–1.42	1.10 (0.87, 1.38)	1.03 (0.79, 1.34)	
	Tertile 1	<LOD–0.52	ref	ref	
**Categorical**					
BCPP	>Median^d^	0.73–54.39	0.84 (0.57, 1.23)	0.88 (0.56, 1.36)	0.14
	≤Median	0.01–0.73	1.15 (0.87, 1.52)	1.18 (0.86, 1.62)	
	<LOD	<LOD	ref	ref	
BCETP	>Median^d^	1.11–207.0	0.82 (0.67, 1.00)	0.76 (0.60, 0.95)	0.052
	≤Median	0.01–1.11	0.84 (0.66, 1.08)	0.83 (0.64, 1.08)	
	<LOD	<LOD	ref	ref	
BBOEP	>Median^d^	0.07–14.89	1.27 (1.08, 1.48)	1.27 (1.07, 1.50)	<0.001
	≤Median	0.01–0.07	0.97 (0.78, 1.19)	0.95 (0.75, 1.19)	
	<LOD	<LOD	ref	ref	
**Detect/Nondetect**					
BMPP	≥LOD		1.30 (0.96, 1.77)	1.34 (0.95, 1.91)	
	<LOD		ref	ref	
BEHP	≥LOD		1.14 (0.89, 1.46)	1.15 (0.83, 1.58)	
	<LOD		ref	ref	
DPRP	≥LOD		0.86 (0.62, 1.20)	0.87 (0.61, 1.26)	
	<LOD		ref	ref	

a Abbreviations: BBOEP, bis­(butoxyethyl) phosphate; BCPP, bis­(1-chloro-2-propyl) phosphate; BCETP, bis­(2-chloroethyl) phosphate; BDCPP, bis­(1,3-dichloro-2-propyl) phosphate; BEHP, bis­(2-ethylhexyl) phosphate; BMPP, bis­(2-methylphenyl) phosphate; BMI, body mass index; CI, confidence interval; DBUP/DIBP, composite of dibutyl phosphate and diisobutyl phosphate; DPHP, di­(2-propylheptyl) phthalate; DPRP, dipropyl phosphate; ECHO, Environmental influences on Child Health Outcomes; LOD, limit of detection; MICE, multiple imputation by chained equations; OPE, organophosphate ester flame retardant and plasticizer; SRS, Social Responsiveness Scale.

b Dilution corrected, untransformed values, μg/L.

c Adjusted for cohort.

d Adjusted MICE models included ECHO cohort, maternal age (continuous), race/ethnicity, prepregnancy BMI, educational attainment, sex, parity, and smoking during pregnancy^; dc^ overall *p*-value of the three-category OPE exposure variable calculated from a Type III Wald test.

e Median of the detectable values.

In sex-stratified models, DPHP exposure was associated with lower odds of autism in males (OR-adj [95% CI]: 0.90 [0.83, 0.98]), but this was not significantly different from the association among females (OR-adj [95% CI]: 0.96 [0.84, 1.10]) (*p*_int_ = 0.21). High exposure to BCPP was associated with lower odds of autism in males (OR-adj [95% CI]: 0.80 [0.65, 0.99]) and higher odds of autism among females (OR-adj [95% CI]: 1.14 [0.47, 2.80]), though the latter estimate was imprecise (*p*_int_ = 0.08) (Table 5). The high-BBOEP exposure category was associated with greater odds of autism in males (OR-adj [95% CI]: 1.30 [1.13, 1.49]), but this association was also not significantly different from that among females (OR-adj [95% CI]: 1.17 [0.68, 2.01]) (*p*_int_ = 0.87). Lastly, the association between detectable (vs nondetectable) DPRP and autism diagnosis differed by sex, with lower odds among females (OR-adj [95% CI]: 0.55 [0.27, 1.10]) and no association among males (OR-adj [95% CI]: 1.04 [0.76, 1.42]) (*p*_int_ = 0.02).

**Table 5:** Associations of OPEs and Autism Clinical Diagnosis in Logistic Models Fitted with Generalized Estimating Equations with Clustering by ECHO Cohort, Stratified by Child Sex^e^

		**Clinical Autism Diagnosis**			
		**Male**	**Female**		
**OPE Analyte**	**Level**	**OR**_**adj**_ **(95% CI)** ^a^ *n* = 1855	**OR**_**adj**_ **(95% CI)** ^a^ *n* = 1825	***P*_interaction_** ^b^	***P*_interaction_** ^c^
**Continuous**					
DBUP/DIBP		1.02 (0.92, 1.13)	1.05 (0.92, 1.21)	0.69	
DPHP		0.90 (0.83, 0.98)	0.96 (0.84, 1.10)	0.21	
BDCPP		0.99 (0.97, 1.02)	1.03 (0.97, 1.09)	0.45	
**Categorical**					
BCPP	>Median^d^	0.80 (0.65, 0.99)	1.14 (0.47, 2.80)	0.083	0.22
	≤Median	1.19 (0.88, 1.62)	1.07 (0.77, 1.48)	0.64	
	<LOD	ref	ref		
BCETP	>Median^d^	0.81 (0.57, 1.13)	0.69 (0.39, 1.23)	0.43	0.21
	≤Median	0.95 (0.69, 1.30)	0.63 (0.38, 1.03)	0.1	
	<LOD	ref	ref		
BBOEP	>Median^d^	1.30 (1.13, 1.49)	1.17 (0.68, 2.01)	0.87	0.97
	≤Median	0.92 (0.74, 1.14)	1.01 (0.65, 1.56)	0.97	
	<LOD	ref	ref		
**Detect/Nondetect**					
BMPP	≥LOD	1.32 (0.99, 1.77)	1.40 (0.82, 2.40)	0.82	
	<LOD	ref	ref		
BEHP	≥LOD	1.17 (0.79, 1.73)	0.96 (0.62, 1.49)	0.48	
	<LOD	ref	ref		
DPRP	≥LOD	1.04 (0.76, 1.42)	0.55 (0.27, 1.10)	0.015	
	<LOD	ref	ref		

a Abbreviations: BBOEP, bis­(butoxyethyl) phosphate; BCPP, bis­(1-chloro-2-propyl) phosphate; BCETP, bis­(2-chloroethyl) phosphate; BDCPP, bis­(1,3-dichloro-2-propyl) phosphate; BEHP, bis­(2-ethylhexyl) phosphate; BMPP, bis­(2-methylphenyl) phosphate; BMI, body mass index; CI, confidence interval; DBUP/DIBP, composite of dibutyl phosphate and diisobutyl phosphate; DPHP, di­(2-propylheptyl) phthalate; DPRP, dipropyl phosphate; ECHO, Environmental influences on Child Health Outcomes; LOD, limit of detection; MICE, multiple imputation by chained equations; OPE, organophosphate ester flame retardant and plasticizer; SRS, Social Responsiveness Scale.

b Adjusted MICE models included ECHO cohort, maternal age (continuous), race/ethnicity, prepregnancy BMI, educational attainment, parity, and smoking during pregnancy.

c The P value was obtained from an interaction model that included both a first-order term for sex and the cross-product term of child sex and OPE analyte added to the adjusted model. We considered P interaction <0.05 to be statistically significant.

d Overall P-value of three-category OPE × sex interaction model using Type III Wald test. We considered P-interaction <0.05 to be statistically significant.

e Median of the detectable values.

### Sensitivity Analyses

Additional adjustment for birth year did not appreciably change results for either SRS or autism models (Table S10). In analyses stratified by SRS version completed, the moderate-BCPP exposure category and the high-BBOEP exposure category remained associated with higher SRS scores on both the preschool (29% of sample) and school-age (71% of sample) forms (Table S11). Detectable (vs nondetectable) levels of DPRP were associated with lower SRS scores on the preschool form but not the school-age form. The results for the SRS were generally unchanged when MARBLES and EARLI, the two cohorts with increased family likelihood of autism, were excluded from the sample (Table S12). One exception was that the moderate-BCETP exposure category showed a stronger association with higher SRS T-scores (β-adj [95% CI]: 037 [0.02, 0.72]) than observed in the primary analysis. The results for BBOEP, BCPP, and BCETP exposure were robust to the exclusion of each ECHO cohort in the leave-one-cohort-out analyses (Figure S6).

When excluding the MARBLES and EARLI cohorts from the autism diagnosis sample, the association between high exposure to BBOEP and greater odds of autism was slightly stronger (OR-adj [95% CI]: 1.41 [1.08, 1.85]), while the association between high exposure to BCETP and lower odds of autism was similar to that in the primary analysis but was no longer statistically significant (OR-adj [95% CI]: 0.69 [0.43, 1.10]) (Table S13). Associations with autism diagnosis were generally robust in leave-one-out analyses (Figure S7). Autism diagnosis results were also consistent in models that restricted the analysis to four cohorts that each contributed ≥15 autism cases (Table S14).

## Discussion

In a large, geographically diverse sample of U.S. children enrolled in 15 cohorts participating in the NIH ECHO Cohort, we found that pregnancy urinary concentrations of some OPEs, specifically exposures to BCPP and BBOEP, were associated with modestly greater scores for child autistic traits. BBOEP exposure was additionally associated with a higher likelihood of clinical diagnosis of autism. These relationships were robust to the exclusion of each ECHO cohort and restriction to general population-based cohorts, excluding two cohorts with increased familial likelihood of autism. Higher exposures to BCPP and BBOEP were also associated with greater scores on the SRS subscales of social communication and interaction difficulties and restricted interests and repetitive behavior as well as with higher likelihood of exceeding the SRS cutoffs for moderate or severe autistic traits. In contrast, high exposure to BCETP was associated with lower odds of autism diagnosis but not SRS scores in the overall sample. We observed stronger associations between BBOEP exposure and SRS scores among males compared with those among females, suggesting potential effect modification by child sex. While BBOEP exposure was also associated with greater odds of an autism diagnosis among males, the estimate was not significantly different from that observed among females.

Our study is the first, to our knowledge, to examine prenatal OPE exposure in relation to autism-related outcomes in a sample largely comprising individuals from the general population. Our findings partially conflict with those from the MARBLES study, which was one of the ECHO cohorts in our sample and is the only previous study to date that has examined OPEs and autism.^29^ MARBLES reported null findings between gestational OPE exposure and autism diagnosis, though their overall sample was smaller (*n* = 277), carried a higher genetic likelihood of familial autism, and had lower detectability of some OPEs than participants in the present study.

While studies examining prenatal OPEs in relation to autism-related outcomes are scarce, our findings expand on several previous studies that have examined other neurodevelopmental end points that show an overlap with autism. BBOEP exposure, which we found to be linearly associated with autistic traits, was previously linked to greater problems with executive functioning in a Norwegian cohort and showed a U-shaped relationship with externalizing problems in the Maternal and Developmental Risks from Environmental and Social Stressors (MADRES) cohort, which was also an ECHO cohort in this analysis.^23,24^ The median concentration of BBOEP in the ECHO cohort (0.05 μg/L) was similar to that in the MADRES cohort (0.04 μg/L); however, comparisons with the Norwegian cohort are challenging due to the higher LOD of their analytical method. Other studies across the US, Norway, and China have reported associations between prenatal exposure to OPEs and increased likelihood of ADHD-like behaviors^16–18^ and reduced cognitive functioning in preschool and older children,^18,22,48^ though the levels of the implicated OPEs—DPHP, DIBP, BDCPP, BMPP, and isopropylphenyl phenyl phosphate (ip-PPP)—were either not significant or not measured (i.e., ip-PPP) in our study. Median urinary concentrations of DPHP and BDCPP, for example, differed in some of these samples relative to the ECHO cohort (0.95 and 0.88 μg/L in 2006–2020, respectively): Concentrations were higher in North Carolina (1.31 and 1.85 μg/L in 2001–2006)^48^ and lower in China (0.29 and 0.10 μg/L in 2014–2016)^22^ and in Norway (0.44 and <LOD of 0.17 μg/L in 2003–2008).^23^ However, two U.S. cohorts in California^18,24^ and the general National Health and Nutrition Examination Survey (NHANES) population in 2013–2014 ^5^ had similar levels of DPHP and BDCPP as our ECHO sample (Figure S8).

BCPP, which showed a potential nonlinear association with autistic traits in our sample, was not measured in most previous studies and has therefore received less attention as a potential neurotoxicant in both epidemiological and toxicological literature. However, evidence is emerging that links early life BCPP exposure to poorer infant neurodevelopment^49^ and neurobehavioral and physiologic changes in fish models.^50–52^ While nonlinear relationships are commonly observed with endocrine-disrupting chemicals, including OPEs, the mechanisms by which BCPP—or potentially other correlated but unmeasured metabolite biomarkers of TCPP^53^—could induce such a relationship remain unclear due to limited knowledge about TCPP’s toxicological pathways. Furthermore, BCETP, which also has limited evidence of developmental toxicity,^54,55^ demonstrated an unexpected inverse association with autism diagnosis in our primary analysis. Unmeasured or residual confounding could contribute to the observed association, as OPE exposures have been linked to socioeconomic, diet, and lifestyle factors.^4,56,57^

Previous studies examining sex differences in the relationship of prenatal OPE exposure and child neurodevelopment have reported mixed results. Our findings—which suggested that BBOEP may be associated with greater autistic traits among males than among females—are consistent with several studies that have observed stronger adverse associations between prenatal OPEs and cognitive development, ADHD, executive functioning, and behavioral problems in males.^16,22–24^ BBOEP specifically was associated with a slightly greater odds of ADHD among males and lower odds among females in the Norwegian Mother, Father, and Child Cohort (MoBa) Study.^16^ However, a handful of other studies, many of which have been relatively small, have found OPEs to be associated with more pronounced adverse associations among females^17,48,58,59^ or no evidence of sex differences.^18^ Toxicological evidence also suggests differential sensitivity to prenatal OPE exposures by sex, though effects appear to vary by neurodevelopmental outcome and animal model. For example, studies report molecular and anatomical changes in brain tissue^60–62^ and anxiety-like behaviors in male rats, hyperactivity in female rats,^63^ and anxiety-like behaviors and reduced social interaction in female prairie voles.^64^ Thus, the potential sex-specific effects of prenatal OPE exposure on child neurodevelopment warrant continued study.

OPEs have been detected in both placental and brain tissues in human and animal samples,^65–67^ suggesting their capacity to permeate the placenta and blood-brain barrier. Toxicological and epidemiological studies have implicated various pathways through which OPEs could plausibly alter child neurodevelopment, potentially in a sexually dimorphic manner. These include disruption of neurotransmitter signaling, promotion of inflammation and oxidative stress, and impairment of neuronal growth and maturation.^11^ Endocrine disruption, particularly of sex and thyroid hormones, has also been reported in epidemiological studies.^68–74^ Prenatal BBOEP exposure, for example, was associated with higher thyroid-stimulating hormone levels in male newborns in the MoBa study.^71^ Furthermore, in vitro studies suggest that TBOEP, BBOEP’s precursor, can interfere with thyroid hormone binding to transporter proteins.^75^ Thyroid hormone disruption has also been linked to shorter gestational length,^76^ which is associated with autism and other neurodevelopmental outcomes.^77,78^ In a previous study in the ECHO cohort, pregnancy urine concentrations of BBOEP were associated with shorter gestations, including higher risk of preterm birth.^12^ Whether thyroid disruption and gestational duration mediate the relationships we observed merits future investigation.

Our study has several strengths, including the large size and sociodemographic and geographic diversity of the ECHO cohort. Although we pooled data across heterogeneous ECHO cohorts, all urine specimens were assayed for OPEs using the same analytical methods at the same laboratory, which reduced measurement errors. We examined both quantitative traits of autism and reported clinical diagnosis, allowing us to examine the consistency of the results across both subclinical and clinical outcomes. However, it should be noted that while high scores on the SRS often correspond with an autism diagnosis, conditions such as ADHD are also correlated with both the SRS and autism^79,80^ and have been linked to OPE exposure. To address the influence of heterogeneity across the ECHO cohorts, including variability in geography and birth years, we implemented generalized estimating equations with clustering by cohort and examined the robustness of the results to the exclusion of individual cohorts and cohorts with increased familial likelihood of autism.

However, there are some limitations. While we included a diverse cross-section of the U.S. pediatric population, each of the ECHO cohorts had differences in their eligibility criteria (e.g., two cohorts enrolled children with an autistic older sibling, and one site enrolled pregnant people who smoked tobacco during pregnancy), and not all ECHO cohorts contributed data to this current analysis. Thus, it will be important to replicate these findings in additional geographic locations and populations where the underlying distributions of risk factors may vary. Since we examined OPEs at only a single time point, we may not have accurately captured typical exposure levels during pregnancy and could not examine critical exposure windows. Due to the body’s rapid metabolism of OPEs, urinary concentrations can fluctuate daily^81^ and some exposures may be episodic, as suggested by their low detectability in our sample. Although sources of everyday exposure to OPEs likely remain stable during pregnancy, concentrations and routes of exposure may vary depending on the temperature or ventilation in the environment, with higher indoor temperatures in homes and cars potentially contributing to greater OPE volatilization and subsequent inhalation exposures in summer months.^82^ It is therefore important to confirm our findings in study samples with multiple measures of OPEs across pregnancy. We also conducted a number of statistical tests across nine biomarkers and two correlated autism-related outcomes, which may have increased the possibility of type I error. However, rather than adjusting for multiple comparisons, which could increase the likelihood of missing subtle but important associations, we followed guidance to base our conclusions on the consistency and robustness of associations across these outcomes and both primary and subanalyses, interpreted within the context of existing literature.^83,84^ Given their low correlations and the fact that only three OPEs had enough detectability to be analyzed continuously, we also did not examine exposure mixtures to understand how these OPEs may interact additively or multiplicatively. This remains an important avenue for future work. Lastly, while we examined a larger set of OPE metabolites than has been included in previous studies, there are still many OPEs in use today that have not been well characterized and that have limited toxicity testing.

## Conclusion

This study offers evidence from a large and diverse sample in the United States that prenatal exposure to some OPEs may be associated with modest increases in autistic traits and the likelihood of autism diagnosis in childhood. These findings were generally consistent across different secondary and sensitivity analyses. Relationships between BBOEP exposure and autism-related outcomes were more pronounced in males than in females, suggesting potential effect modification by the child sex. These findings contribute to the growing body of evidence suggesting that OPEs, exposure to which has become ubiquitous among pregnant people and children, are potential developmental neurotoxicants of concern.

## Supplementary Material





## Notes

Full list of authors (XLSX)
Tables describing the cohorts, variable information in ECHO study variable harmonization, descriptive characteristics of participants, overlap between autism diagnosisand SRS T-scores, demographic characteristics of participants, distributions of OPEs, associations of OPEs and SRS raw scores, associations of OPEs and T-scores on SRS subscales, associations of OPEs and SRS clinical cutpoints, birth year adjustment sensitivity analysis, associations between OPEs and SRS total T-score, associations of OPEs and SRS T-score, associations of OPEs and ASD diagnoses, associations of OPEs and autism diagnoses; figures of flow chart of included participants, relationship between pregnancy urine concentrations of OPE exposure and autism-related outcomes, boxplots of OPE analytes and analyte distributions, Spearman correlations, coefficient plots, comparison of DPHP and BDCPP concentrations (DOCX)
