# Supplementary material for: Exposure to Organophosphate Ester Flame Retardants and Plasticizers during Pregnancy and Autism-Related Outcomes in the ECHO Cohort
Source: Environ Health Perspect. 2026 May 7;134(4):423–37. doi: 10.1021/EHP.6c00247 (PMC13445271; doi:10.1021/EHP.6c00247)
Supplement: Supplementary file 1 [file hp6c00247_si_002.docx]

**Supplementary Material**

**Table S1**. Description of the cohorts included

**Table S2.** Source of variable information in ECHO study variable harmonization

**Table S3**. Descriptive characteristics of participants contributing to analyses, by NIH ECHO site (SRS sample)

**Table S4.** Overlap between autism diagnosis and SRS T-scores among participants with both clinical history information and SRS assessments.

**Table S5**. Demographic characteristics of participants from 15 ECHO cohorts by study sample inclusion status.

**Table S6.** Distributions of OPEs (dilution corrected, untransformed values, μg/L) and % below the limit of detection (LOD) in A) pooled sample, B) SRS Sample, and C) Autism Diagnosis sample.

**Table S7**. Associations of OPEs and SRS raw scores in linear models fitted with generalized estimating equations with clustering by ECHO cohort.

**Table S8**. Associations of OPEs and T-scores on SRS subscales in linear models fitted with generalized estimating equations with clustering by ECHO cohort.

**Table S9**. Associations of OPEs and SRS clinical cutpoints in logistic models fitted with generalized estimating equations with clustering by ECHO cohort.

**Table S10**. Birth year adjustment sensitivity analysis of associations of OPEs and SRS T-scores and Clinical autism diagnosis in models fitted with generalized estimating equations.

**Table S11**. Associations between OPEs and SRS total T-score in linear models fitted with generalized estimating equations with clustering by ECHO cohort, stratified by SRS-2 form completed

**Table S12**. Associations of OPEs and SRS T-score in linear models fitted with generalized estimating equations with clustering by ECHO cohort, after excluding MARBLES and EARLI

**Table S13**. Associations of OPEs and ASD diagnoses in logistic models fitted with generalized estimating equations with clustering by ECHO cohort, after excluding MARBLES and EARLI

**Table S14.** Associations of OPEs and autism diagnoses in logistic models fitted with generalized estimating equations, restricted to 4 cohorts with >= 15 autism cases: Marbles, EARLI, Candle, and TIDES

**Figure S1:** Flow chart of included participants

**Figure S2**. Direct acyclic graph of the relationship between pregnancy urine concentrations of organophosphate ester (OPE) exposure and autism-related outcomes

**Figure S3**. Boxplots of OPE analytes (log2-transformed, dilution corrected) in the pooled ECHO sample

**Figure S4**. Boxplots of OPE analyte distributions (log2-transformed, dilution corrected) by ECHO cohort

**Figure S5**. Spearman correlations on all OPEs, in the full sample, and by SRS or autism cohort, imputed, dilution corrected.

**Figure S6**. Coefficient plots of leave-one-out results for SRS total T-score

**Figure S7**. Coefficient plots of leave-one-out results for clinical autism spectrum disorder (ASD) diagnosis

**Figure S8**: Comparison of DPHP and BDCPP concentrations across studies

**Table S1**. Description of the cohorts included

| **Cohort name** | **N** | **Cohort features** | **Eligibility criteria** | **Location** |
| --- | --- | --- | --- | --- |
| Healthy Start^1^ | 204 | Pregnant women recruited from obstetrics clinics at a university hospital in the Denver, Colorado metropolitan area and their offspring. | Eligible women were ≥16 years old, had singleton pregnancies, and had no history of previous stillbirth or extremely preterm birth, and no self-reported history of diabetes, cancer, asthma treated with steroids, or psychiatric conditions. | Denver, Colorado |
| Pregnancy Environment and Lifestyle Study (PETALS)^2^ | 219 | Women identified as pregnant via hospital records from 4 California hospitals recruited before 10 weeks gestation via telephone and their offspring | Eligible women were aged 18–45 years, able to provide informed consent in English, had singleton pregnancies, and did not have a pre-conception history of diabetes, cancer, hepatitis C, or liver cirrhosis. | Northern California |
| Maternal and Developmental Risks from Environmental and Social Stressors (MADRES)^3^ | 106 | Pregnant women recruited from 4 OB/GYN clinics serving low income populations in Los Angeles, CA and their offspring. | Eligible mothers were ≥18 years old, had singleton pregnancies, were fluent speakers of English or Spanish, were not incarcerated, were not HIV positive, and had no physical, mental or cognitive disability that would prevent participation or informed consent. | Los Angeles, California |
| New Hampshire Birth Cohort Study (NHBCS)^4^ | 876 | Pregnant women recruited from prenatal clinics in the rural New Hampshire and their offspring | Eligible women were 18–45 years old,  spoke English, had singleton pregnancies, and used a private, unregulated water system (e.g., private well) at home. | New Hampshire |
| Conditions Affecting Neurocognitive Development and Learning in Early Childhood (CANDLE)^5^ | 1177 | A majority African American sample of mother-child dyads recruited in pregnancy in Memphis, TN from safety net obstetrical clinics and local OB/GYN partners, television and radio advertisements, and directed mailings | Eligibility criteria included planning to deliver at one of five hospitals in Memphis, aged between 16–40 years,  residence in Shelby County, TN, and having a low medical risk pregnancy. Eligible women had English fluency, had a singleton pregnancy, and were between 16-28 weeks gestation at enrollment. | Memphis, Tennessee |
| Vitamin C to Decrease the Effects of Smoking in Pregnancy on Infant Lung Function (VCSIP)^6^ | 98 | Pregnant women who smoke receiving prenatal care at 3 clinics across the US (Indiana, Washington, Oregon) and their offspring. | Eligible women were at least 15 years old, pregnant with randomization prior to 22 weeks of gestation, cigarette smoker, singleton gestation, English speaking, and receiving prenatal care at clinics delivering at specific locations. | Indiana, Washington, Oregon |
| University of California - Markers of Autism Risk in Babies – Learning Early Signs (MARBLES)^7^ | 249 | Pregnant women who have a biological child diagnosed with autism spectrum disorder (ASD) and the offspring of the current pregnancy, recruited from the California Department of Developmental Services list of families receiving services for a child with ASD. | Eligible women a) had one or more  biological child(ren) or first-degree  relatives with ASD or the gestating child had an older half-sibling with ASD; b) was ≥18 years old; d) was proficient in English and the gestating child would be taught to speak English; e) lived within 2.5 hours of the Davis/Sacramento region. | Northern California |
| Early Autism Risk  Longitudinal  Investigation  (EARLI)^8^ | 91 | Pregnant women with a biological  child with autism spectrum  disorder (ASD) and the offspring  of the current pregnancy,  recruited from local community  events, autism conferences, direct  mail via autism service providers, and online and social media. | Eligible women had a biological child who had been diagnosed with an ASD, were competent to communicate in English (or, at two sites, in Spanish), were 18 years of age or older, lived within two hours of a study clinic, and were no more than 28 weeks pregnant. | California, Pennsylvania, Maryland |
| Rochester^9^ | 174 | Women recruited during the first trimester of pregnancy from clinics in Rochester, NY serving high psychosocial risk populations, oversampled for psychosocial stress. | Eligible women were ≥18 years old, had singleton pregnancies, and had no known substance abuse issues or history of psychotic illness, no major endocrine disorder, high-risk health condition, or significant obstetric concern at baseline. | Rochester, New York |
| Magee | 63 | Women recruited during the first trimester of pregnancy from clinics in Pittsburgh, PA serving high psychosocial risk populations, oversampled for childhood trauma, and their offspring. | Eligibility criteria for mothers at  enrollment: Inclusion criteria were a) ≥18 years age, b) singleton, intrauterine pregnancy, c) ≤16 weeks gestation at recruitment, and d) fluency in English. Exclusion criteria were a) presence of congenital mal-formations or fetal  chromosomal abnormalities; b) presence of any conditions that may dysregulate endocrine, immune or oxidative state or systemic inflammation, such as autoimmune disorders requiring chronic systemic steroid or immunomodulator use. | Pittsburgh, Pennsylvania |
| Fair Start^10^ | 68 | Dominican or African American low-income mothers and their offspring recruited at OB/GYN clinics in New York, NY. | Eligible mothers were fluent in English  or Spanish, had singleton pregnancies,  and were ≥18 years of age. | New York, New York |
| Illinois Kids Development Study (IKIDS)^11^ | 149 | Pregnant women recruited from 2 OB/GYN clinics in central Illinois and their offspring | Eligible mothers were 18–40 years old, had singleton pregnancies, were not in a high risk pregnancy, were fluent in English, and were not planning to leave the area before the child’s first birthday. | Illinois |
| Chemicals in our Bodies (CIOB)^12^ | 160 | Pregnant women recruited from 2 hospitals in San Francisco, CA and their offspring. | Eligible mothers were fluent in English or Spanish, had singleton births, were ≥18 years of age, and had no major pregnancy complications. | San Francisco, California |
| The NYU Children’s Health and Environment Study (NYU CHES)^13^ | 45 | Pregnant women recruited from prenatal clinics at NYU Langone Medical Center study sites. | Eligible women were ≥18 years old, <18 weeks gestation, not carrying a medically threatened pregnancy, and intending to deliver at one of three NYU-affiliated hospitals. | New York, New York |
| The Infant  Development and  the Environment  Study (TIDES) ^14^ | 480 | Pregnant women recruited during  the first trimester from prenatal  clinics in Minnesota, New York, California, and Washington, and  their offspring. | Eligible women were ≥18 years old, with a non-medically threatened singleton pregnancy and able to communicate in English. | Minnesota, New York,  California, and Washington |

**Table S2.** Source of variable information in ECHO study variable harmonization

| **Variables** | **Questionnaire or Medical Record Abstraction** |
| --- | --- |
| **Maternal age** | Questionnaire |
| **Maternal education** | Questionnaire |
| **Maternal race** | Questionnaire |
| **Parity** | Questionnaire, Medical Record Abstraction |
| **Pre-pregnancy BMI** | Questionnaire, Medical Record Abstraction |
| **Tobacco use during pregnancy** | Questionnaire, Medical Record Abstraction |
| **Child Sex** | Questionnaire |
| **Year of birth** | Questionnaire |
| **Social Responsiveness Scale** | Questionnaire |
| **Clinical ASD diagnosis** | Questionnaire or Medical Record Abstraction |

**Table S3**. Descriptive characteristics of participants contributing to analyses, by NIH ECHO cohort (SRS sample)

|  | **Healthy Start** | **PETALS** | | **MADRES** | **NHBCS** | **CANDLE** | **VCSIP** | **MARBLES** |
| --- | --- | --- | --- | --- | --- | --- | --- | --- |
|  | **(n=198)** | **(n=190)** | | **(n=80)** | **(n=840)** | **(n=755)** | **(n=85)** | **(n=119)** |
| **Maternal age** |  |  | |  |  |  |  |  |
| 1. 16–24 | 23 (11.6%) | 5 (2.6%) | | 16 (20.0%) | 50 (6.0%) | 258 (34.2%) | 25 (29.4%) | <5 (<1.0%) |
| 2. 25–29 | 48 (24.2%) | 41 (21.6%) | | 22 (27.5%) | 219 (26.1%) | 229 (30.3%) | 25 (29.4%) | <15 (<15.0%) |
| 3. 30–34 | 81 (40.9%) | 81 (42.6%) | | 25 (31.3%) | 337 (40.1%) | 176 (23.3%) | 24 (28.2%) | 37 (31.1%) |
| 4. 35–47 | 46 (23.2%) | 63 (33.2%) | | 17 (21.3%) | 234 (27.9%) | 92 (12.2%) | 11 (12.9%) | 67 (56.3%) |
| **Maternal education** |  |  | |  |  |  |  |  |
| 1. Up to high school degree, GED, or Equivalent | 23 (11.6%) | 18 (9.5%) | | 36 (45.0%) | 71 (8.5%) | 418 (55.4%) | 46 (54.1%) | 10 (8.4%) |
| 2. Some college, no degree | 34 (17.2%) | 64 (33.7%) | | 19 (23.8%) | 129 (15.4%) | 72 (9.5%) | 34 (40.0%) | 41 (34.5%) |
| 3. Bachelor's degree. | 69 (34.8%) | 67 (35.3%) | | 11 (13.8%) | 300 (35.7%) | 164 (21.7%) | <5 (<5.0%) | 43 (36.1%) |
| 4. Master and above | 72 (36.4%) | 40 (21.1%) | | 8 (10.0%) | 241 (28.7%) | 100 (13.2%) | 0 (0.0%) | 21 (17.6%) |
| 5. Missing | 0 (0.0%) | 1 (0.5%) | | 6 (7.5%) | 99 (11.8%) | 1 (0.1%) | <5 (<5.0%) | 4 (3.4%) |
| **Maternal race** |  |  | |  |  |  |  |  |
| 1. Hispanic All | 26 (13.1%) | 79 (41.6%) | | 66 (82.5%) | 17 (2.0%) | 9 (1.2%) | <5 (<5.0%) | 33 (27.7%) |
| 2. Non-Hispanic Black | 11 (5.6%) | 8 (4.2%) | | 5 (6.3%) | <5 (<1.0%) | 454 (60.1%) | 11 (12.9%) | <10 (<5.0%) |
| 3. Non-Hispanic Asian | 8 (4.0%) | 55 (28.9%) | | <5 (<5.0%) | <10 (<1.0%) | 5 (0.7%) | 0 (0.0%) | 15 (12.6%) |
| 4. Non-Hispanic Other (pacific islander, American Indian, multi-race and other) | <5 (<5.0%) | 11 (5.8%) | | 0 (0.0%) | 17 (2.0%) | 31 (4.1%) | 9 (10.6%) | <5 (<5.0%) |
| 5. Non-Hispanic White | 148 (74.7%) | 37 (19.5%) | | 7 (8.8%) | 786 (93.6%) | 256 (33.9%) | 62 (72.9%) | 61 (51.3%) |
| 6. Missing/Unknown | <5 (<5.0%) | 0 (0.0%) | | <5 (<5.0%) | 12 (1.4%) | 0 (0.0%) | <5 (<5.0%) | 0 (0.0%) |
| **Parity** |  |  | |  |  |  |  |  |
| 1. Nulliparous | 79 (39.9%) | 71 (37.4%) | | 28 (35.0%) | 383 (45.6%) | 320 (42.4%) | 28 (32.9%) | 0 (0.0%) |
| 2. Multiparous | 119 (60.1%) | 119 (62.6%) | | 45 (56.3%) | 446 (53.1%) | 435 (57.6%) | 57 (67.1%) | <120 (<97%) |
| 3. Missing | 0 (0.0%) | 0 (0.0%) | | 7 (8.8%) | 11 (1.3%) | 0 (0.0%) | 0 (0.0%) | <5 |
| **Table S3 (cont)** | **Healthy Start** | **PETALS** | | **MADRES** | **NHBCS** | **CANDLE** | **VCSIP** | **MARBLES** |
| **Pre-pregnancy BMI** |  |  | |  |  |  |  |  |
| 1. <18.5 | 5 (2.5%) | <5 (<5.0%) | | 0 (0.0%) | 24 (2.9%) | 35 (4.6%) | <5 (<5.0%) | <5 (<5.0%) |
| 2. 18.5–24.9 | 108 (54.5%) | 68 (35.8%) | | 25 (31.3%) | 480 (57.1%) | 293 (38.8%) | 19 (22.4%) | 46 (38.7%) |
| 3. 25.0–29.9 | 51 (25.8%) | 50 (26.3%) | | 29 (36.3%) | 203 (24.2%) | 178 (23.6%) | 25 (29.4%) | 29 (24.4%) |
| 4. >=30.0 | 34 (17.2%) | 69 (36.3%) | | 26 (32.5%) | 124 (14.8%) | 247 (32.7%) | 39 (45.9%) | 42 (35.3%) |
| 5. Missing | 0 (0.0%) | <5 (<5.0%) | | 0 (0.0%) | 9 (1.1%) | 2 (0.3%) | <5 (<5.0%) | <5 (<5.0%) |
| **Tobacco use during pregnancy** |  |  | |  |  |  |  |  |
| 1. No | 187 (94.4%) | 187 (98.4%) | | 79 (98.8%) | 792 (94.3%) | 688 (91.1%) | 0 (0.0%) | 115 (96.6%) |
| 2. Yes | 11 (5.6%) | <5 (<5.0%) | | 0 (0.0%) | 46 (5.5%) | 66 (8.7%) | 85 (100%) | 0 (0.0%) |
| 3. Missing | 0 (0.0%) | <5 (<5.0%) | | 1 (1.3%) | 2 (0.2%) | 1 (0.1%) | 0 (0.0%) | 4 (3.4%) |
| **Child Sex** |  |  | |  |  |  |  |  |
| 1. Female | 94 (47.5%) | 88 (46.3%) | | 43 (53.8%) | 422 (50.2%) | 382 (50.6%) | 43 (50.6%) | 49 (41.2%) |
| 2. Male | 104 (52.5%) | 102 (53.7%) | | 37 (46.3%) | 418 (49.8%) | 373 (49.4%) | 42 (49.4%) | 70 (58.8%) |
| **Year of birth** |  |  | |  |  |  |  |  |
| 1. 2006–2010 | 0 (0.0%) | 0 (0.0%) | | 0 (0.0%) | 179 (21.3%) | 648 (85.8%) | 0 (0.0%) | 37 (31.1%) |
| 2. 2011–2015 | 198 (100%) | 117 (61.6%) | | 0 (0.0%) | 584 (69.5%) | 107 (14.2%) | 85 (100%) | 65 (54.6%) |
| 3. 2016–2020 | 0 (0.0%) | 73 (38.4%) | | 80 (100%) | 77 (9.2%) | 0 (0.0%) | 0 (0.0%) | 17 (14.3%) |
| **SRS Second Edition (SRS-2) Version** |  |  | |  |  |  |  |  |
| SRS-2 Preschool  (ages 2.5–4.5y) | 0 (0.0%) | 0 (0.0%) | | 0 (0.0%) | 228 (27.1%) | 0 (0.0%) | 0 (0.0%) | 19 (16.0%) |
| SRS-2 School-Age  (ages 4–18y) | 198 (100%) | 190 (100%) | | 80 (100%) | 612 (72.9%) | 755 (100%) | 85 (100%) | 100 (84.0%) |
| **SRS form** |  |  | |  |  |  |  |  |
| Full | 198 (100%) | 190 (100%) | | 80 (100%) | 840 (100%) | 215 (28.5%) | 85 (100%) | 119 (100%) |
| Short | 0 (0.0%) | 0 (0.0%) | | 0 (0.0%) | 0 (0.0%) | 540 (71.5%) | 0 (0.0%) | 0 (0.0%) |
| **Clinical ASD diagnosis** |  |  | |  |  |  |  |  |
| 1. No | 190 (96.0%) | 181 (95.3%) | | 60 (75.0%) | 467 (55.6%) | 729 (96.6%) | 82 (96.5%) | 90 (75.6%) |
| 2. Yes | <5 (<5.0%) | 9 (4.7%) | | <5 (<10.0%) | <5 (<1.0%) | 24 (3.2%) | <5 (<5.0%) | 28 (23.5%) |
| 3. Missing | <10 (<5.0%) | 0 (0.0%) | | <20 (<20.0%) | <380 (<45.0%) | 2 (0.3%) | <5 (<5.0%) | 1 (0.8%) |
| **Table S3 (cont)** | **Healthy Start** | **PETALS** | | **MADRES** | **NHBCS** | **CANDLE** | **VCSIP** | **MARBLES** |
| **Total SRS raw score** |  |  | |  |  |  |  |  |
| Mean (SD) | 28.1 ± 18.0 | 32.4 ± 18.4 | | 39.7 ± 21.1 | 27.6 ± 17.9 | 33.3 ± 21.4 | 43.8 ± 26.0 | 37.1 ± 28.5 |
| Median (Q1–Q3) | 25.0 (15.0–37.0) | 29.5 (19.0–41.0) | | 36.0 (24.5–48.5) | 24.0 (16.0–35.0) | 28.0 (18.0–44.0) | 34.0 (26.0–59.0) | 27.0 (16.0–51.0) |
| Min-Max | 1.0–120.0 | 4.0–124.0 | | 9.0–122.0 | 1.0–133.0 | 1.0–156.0 | 6.0–130.0 | 4.0–136.0 |
| **Total SRS T-score** |  |  | |  |  |  |  |  |
| Mean (SD) | 48.6 ± 7.2 | 50.4 ± 7.4 | | 53.5 ± 8.6 | 47.4 ± 7.4 | 50.8 ± 8.8 | 55.2 ± 10.8 | 51.5 ± 11.4 |
| Median (Q1–Q3) | 47.0 (44.0–52.0) | 49.0 (45.0–54.0) | | 52.5 (47.5–58.0) | 46.0 (43.0–50.0) | 49.0 (45.0–55.0) | 52.0 (48.0–61.0) | 48.0 (43.0–58.0) |
| Min-Max | 38.0-84.0 | 38.0-86.0 | | 40.0-88.0 | 35.0-93.0 | 38.0-99.0 | 39.0-93.0 | 39.0-95.0 |
| **Child's age at SRS** |  |  | |  |  |  |  |  |
| Mean (SD) | 8.2 ± 0.5 | 4.3 ± 0.5 | | 4.4 ± 0.5 | 5.7 ± 2.3 | 11.5 ± 1.3 | 7.0 ± 1.1 | 8.0 ± 3.3 |
| Median (Q1–Q3) | 8.0 (8.0–8.0) | 4.0 (4.0–4.0) | | 4.0 (4.0–5.0) | 5.0 (3.0–7.0) | 11.0 (10.0–12.0) | 7.0 (6.0–8.0) | 8.0 (6.0–11.0) |
|  |  |  | |  |  |  |  |  |
|  | **EARLI** | **Rochester** | | **Magee** | **Fair Start** | **IKIDS** | **CIOB** | **NYU CHES** |
|  | (n=52) | (n=133) | | (n=54) | (n=39) | (n=147) | (n=96) | (n=41) |
| **Maternal age** |  |  | |  |  |  |  |  |
| 1. 16-24 | <5 (<1.0%) | <5 (<5.0%) | | <5 (<5.0%) | 11 (28.2%) | <5 (<5.0%) | <5 (<5.0%) | 8 (19.5%) |
| 2. 25-29 | <10 (<10.0%) | 36 (27.1%) | | <15 (<30.0%) | 11 (28.2%) | 38 (25.9%) | <10 (<10.0%) | 10 (24.4%) |
| 3. 30-34 | 23 (44.2%) | 60 (45.1%) | | 22 (40.7%) | 12 (30.8%) | 80 (54.4%) | 39 (40.6%) | 6 (14.6%) |
| 4. 35-47 | 23 (44.2%) | <35 (<25.0%) | | 16 (29.6%) | 5 (12.8%) | <30 (20.0%) | 48 (50.0%) | 17 (41.5%) |
| **Maternal education** |  |  | |  |  |  |  |  |
| 1. Up to high school degree, GED, or Equivalent | 8 (15.4%) | 21 (15.8%) | | 6 (11.1%) | 19 (48.7%) | 0 (0.0%) | 13 (13.5%) | 24 (58.5%) |
| 2. Some college, no degree | 17 (32.7%) | 13 (9.8%) | | 11 (20.4%) | 12 (30.8%) | 20 (13.6%) | <5 (<5.0%) | <5 (<5.0%) |
| 3. Bachelor's degree. | 12 (23.1%) | 39 (29.3%) | | 14 (25.9%) | 7 (17.9%) | 49 (33.3%) | 24 (25.0%) | 7 (17.1%) |
| 4. Master and above | 13 (25.0%) | 49 (36.8%) | | 20 (37.0%) | 0 (0.0%) | 78 (53.1%) | 56 (58.3%) | 7 (17.1%) |
| 5. Missing | 2 (3.8%) | 11 (8.3%) | | 3 (5.6%) | 1 (2.6%) | 0 (0.0%) | <5 (<5.0%) | <5 (<5.0%) |
| **Table S3 (cont)** | **EARLI** | **Rochester** | | **Magee** | **Fair Start** | **IKIDS** | **CIOB** | **NYU CHES** |
| **Maternal race** |  |  | |  |  |  |  |  |
| 1. Hispanic All | 10 (19.2%) | 10 (7.5%) | | <5 (<5.0%) | 38 (97.4%) | 6 (4.1%) | 24 (25.0%) | 28 (68.3%) |
| 2. Non-Hispanic Black | <10 (<15.0%) | 21 (15.8%) | | 12 (22.2%) | <5 (<5.0%) | <5 (<5.0%) | <5 (<5.0%) | <5 (<5.0%) |
| 3. Non-Hispanic Asian | 7 (13.5%) | <5 (<5.0%) | | 0 (0.0%) | 0 (0.0%) | 7 (4.8%) | 21 (21.9%) | 5 (12.2%) |
| 4. Non-Hispanic Other (pacific islander, American Indian, multi-race and other) | <5 (<5.0%) | <5 (<5.0%) | | <10 (<10.0%) | 0 (0.0%) | <5 (<5.0%) | 0 (0.0%) | 0 (0.0%) |
| 5. Non-Hispanic White | 28 (53.8%) | 95 (71.4%) | | 36 (66.7%) | 0 (0.0%) | 127 (86.4%) | 46 (47.9%) | 7 (17.1%) |
| 6. Missing/Unknown | 0 (0.0%) | <5 (<5.0%) | | 0 (0.0%) | <5 (<5.0%) | 0 (0.0%) | <5 (<5.0%) | <5 (<5.0%) |
|  |  |  | |  |  |  |  |  |
| **Parity** |  |  | |  |  |  |  |  |
| 1. Nulliparous | 6 (11.5%) | 38 (28.6%) | | 15 (27.8%) | <5 (<10.0%) | 84 (57.1%) | <90 (<90%) | 19 (46.3%) |
| 2. Multiparous | <49 (<87%) | 95 (71.4%) | | 25 (46.3%) | 12 (30.8%) | 63 (42.9%) | 9 (9.4%) | 22 (53.7%) |
| 3. Missing | <5 | 0 (0.0%) | | 14 (25.9%) | <30 (<65.0%) | 0 (0.0%) | <5 | 0 (0.0%) |
| **Pre-pregnancy BMI** |  |  | |  |  |  |  |  |
| 1. <18.5 | <5 (<5.0%) | <5 (<5.0%) | | <5 (<10.0%) | 0 (0.0%) | 5 (3.4%) | 5 (5.2%) | <5 (<5.0%) |
| 2. 18.5–24.9 | 17 (32.7%) | 53 (39.8%) | | 20 (37.0%) | 19 (48.7%) | 69 (46.9%) | 52 (54.2%) | 19 (46.3%) |
| 3. 25.0–29.9 | 10 (19.2%) | 41 (30.8%) | | 10 (18.5%) | 12 (30.8%) | 32 (21.8%) | 14 (14.6%) | 9 (22.0%) |
| 4. >=30.0 | 24 (46.2%) | 33 (24.8%) | | 11 (20.4%) | 7 (17.9%) | 41 (27.9%) | 6 (6.3%) | 9 (22.0%) |
| 5. Missing | <5 (<5.0%) | <10 (<5.0%) | | <15 (<20.0%) | 1 (2.6%) | 0 (0.0%) | 19 (19.8%) | <5 (<10.0%) |
| **Tobacco use during pregnancy** |  |  | |  |  |  |  |  |
| 1. No | 49 (94.2%) | 0 (0.0%) | | 17 (31.5%) | 38 (97.4%) | 143 (97.3%) | 80 (83.3%) | 38 (92.7%) |
| 2. Yes | 0 (0.0%) | 0 (0.0%) | | 6 (11.1%) | <5 (<5.0%) | <5 (<5.0%) | 0 (0.0%) | <5 (<5.0%) |
| 3. Missing | 3 (5.8%) | 133 (100%) | | 31 (57.4%) | <5 (<5.0%) | <5 (<5.0%) | 16 (16.7%) | <5 (<5.0%) |
| **Child Sex** |  |  | |  |  |  |  |  |
| 1. Female | 22 (42.3%) | 61 (45.9%) | | 24 (44.4%) | 13 (33.3%) | 71 (48.3%) | 45 (46.9%) | 19 (46.3%) |
| 2. Male | 30 (57.7%) | 72 (54.1%) | | 30 (55.6%) | 26 (66.7%) | 76 (51.7%) | 51 (53.1%) | 22 (53.7%) |
|  |  |  | |  |  |  |  |  |
|  |  |  | |  |  |  |  |  |
| **Table S3 (cont)** | **EARLI** | **Rochester** | | **Magee** | **Fair Start** | **IKIDS** | **CIOB** | **NYU CHES** |
| Year of birth |  |  | |  |  |  |  |  |
| 1. 2006–2010 | 24 (46.2%) | <5 (<5.0%) | | 0 (0.0%) | 0 (0.0%) | 0 (0.0%) | 0 (0.0%) | 0 (0.0%) |
| 2. 2011–2015 | 28 (53.8%) | <5 (<5.0%) | | 0 (0.0%) | 7 (17.9%) | 79 (53.7%) | 29 (30.2%) | 0 (0.0%) |
| 3. 2016–2020 | 0 (0.0%) | 130 (97.7%) | | 54 (100%) | 32 (82.1%) | 68 (46.3%) | 67 (69.8%) | 41 (100%) |
| **SRS Second Edition (SRS-2) Version** |  |  | |  |  |  |  |  |
| SRS-2 Preschool (ages 2.5–4.5y) | 0 (0.0%) | 133 (100%) | | 54 (100%) | 26 (66.7%) | 87 (59.2%) | 85 (88.5%) | 19 (46.3%) |
| SRS-2 School-Age (ages 4–18y) | 52 (100%) | 0 (0.0%) | | 0 (0.0%) | 13 (33.3%) | 60 (40.8%) | 11 (11.5%) | 22 (53.7%) |
| Missing |  |  | |  |  |  |  |  |
| **SRS form** |  |  | |  |  |  |  |  |
| Full | 52 (100%) | 133 (100%) | | 54 (100%) | 39 (100%) | 147 (100%) | 96 (100%) | 41 (100%) |
| Short | 0 (0.0%) | 0 (0.0%) | | 0 (0.0%) | 0 (0.0%) | 0 (0.0%) | 0 (0.0%) | 0 (0.0%) |
| **Clinical ASD diagnosis** |  |  | |  |  |  |  |  |
| 1. No | 32 (61.5%) | 87 (65.4%) | | 41 (75.9%) | 36 (92.3%) | 140 (95.2%) | 84 (87.5%) | 40 (97.6%) |
| 2. Yes | 20 (38.5%) | 0 (0.0%) | | <5 (10.0%) | <5 (<5.0%) | <5 (<5.0%) | 5 (5.2%) | 0 (0.0%) |
| 3. Missing | 0 (0.0%) | 46 (34.6%) | | <15 (20.0%) | <5 (<5.0%) | <5 (<5.0%) | 7 (7.3%) | 1 (2.4%) |
| **Total SRS raw score** |  |  | |  |  |  |  |  |
| Mean (SD) | 43.5 ± 33.8 | 31.0 ± 17.7 | | 39.4 ± 24.2 | 38.3 ± 25.6 | 28.6 ± 17.7 | 29.3 ± 18.9 | 35.0 ± 16.5 |
| Median (Q1–Q3) | 29.5 (21.5–62.0) | 26.0 (20.0–40.0) | | 34.0 (22.0–50.0) | 33.0 (24.0–49.0) | 25.0 (18.0–37.0) | 25.0 (17.0–35.5) | 31.0 (23.0–44.0) |
| Min-Max | 5.0–131.0 | 6.0–122.0 | | 0.0–108.0 | 1.0–123.0 | 4.0–101.0 | 5.0–113.0 | 11.0–87.0 |
| **Total SRS T-score** |  |  | |  |  |  |  |  |
| Mean (SD) | 54.7 ± 13.5 | 45.9 ± 6.8 | | 49.1 ± 9.3 | 50.2 ± 10.4 | 46.6 ± 7.5 | 45.6 ± 7.5 | 49.6 ± 6.8 |
| Median (Q1–Q3) | 49.0 (45.5–62.5) | 44.0 (42.0–49.0) | | 47.0 (42.0–53.0) | 47.0 (44.0–54.0) | 45.0 (42.0–49.0) | 44.0 (40.0–48.0) | 49.0 (45.0–53.0) |
| Min-Max | 39.0–89.0 | 36.0–81.0 | | 34.0–76.0 | 34.0–86.0 | 36.0–75.0 | 36.0–78.0 | 39.0–67.0 |
| Child's age at SRS |  |  | |  |  |  |  |  |
| Mean (SD) | 9.1 ± 0.9 | 3.0 ± 0.0 | | 3.3 ± 0.5 | 4.6 ± 1.8 | 4.8 ± 2.0 | 3.7 ± 1.3 | 4.4 ± 0.5 |
| Median (Q1–Q3) | 9.0 (9.0–10.0) | 3.0 (3.0–3.0) | | 3.0 (3.0–4.0) | 4.0 (3.0–6.0) | 4.0 (3.0–7.0) | 3.0 (3.0-4.0) | 4.0 (4.0-5.0) |
| **Table S3 (cont)** | **TIDES** | |  |  |  |  |  |  |
|  | **(n=459)** | |  |  |  |  |  |  |
| **Maternal age** |  | |  |  |  |  |  |  |
| 1. 16–24 | 50 (10.9%) | |  |  |  |  |  |  |
| 2. 25–29 | 96 (20.9%) | |  |  |  |  |  |  |
| 3. 30–34 | 154 (33.6%) | |  |  |  |  |  |  |
| 4. 35–47 | 159 (34.6%) | |  |  |  |  |  |  |
| **Maternal education** |  | |  |  |  |  |  |  |
| 1. Up to high school degree, GED, or Equivalent | 64 (13.9%) | |  |  |  |  |  |  |
| 2. Some college, no degree | 54 (11.8%) | |  |  |  |  |  |  |
| 3. Bachelor's degree. | 143 (31.2%) | |  |  |  |  |  |  |
| 4. Master and above | <200 (<45%) | |  |  |  |  |  |  |
| 5. Missing | <5 | |  |  |  |  |  |  |
| **Maternal race** |  | |  |  |  |  |  |  |
| 1. Hispanic All | 42 (9.2%) | |  |  |  |  |  |  |
| 2. Non-Hispanic Black | 52 (11.3%) | |  |  |  |  |  |  |
| 3. Non-Hispanic Asian | 23 (5.0%) | |  |  |  |  |  |  |
| 4. Non-Hispanic Other (pacific islander, American Indian, multi-race and other) | 32 (7.0%) | |  |  |  |  |  |  |
| 5. Non-Hispanic White | 310 (67.5%) | |  |  |  |  |  |  |
| 6. Missing/Unknown | 0 (0.0%) | |  |  |  |  |  |  |
|  |  | |  |  |  |  |  |  |
| **Parity** |  | |  |  |  |  |  |  |
| 1. Nulliparous | 256 (55.8%) | |  |  |  |  |  |  |
| 2. Multiparous | 197 (42.9%) | |  |  |  |  |  |  |
| 3. Missing | 6 (1.3%) | |  |  |  |  |  |  |
|  |  | |  |  |  |  |  |  |
|  |  | |  |  |  |  |  |  |
|  |  | |  |  |  |  |  |  |
| **Table S3 (cont)** | **TIDES** | |  |  |  |  |  |  |
| **Pre-pregnancy BMI** |  | |  |  |  |  |  |  |
| 1. <18.5 | 5 (1.1%) | |  |  |  |  |  |  |
| 2. 18.5–24.9 | 212 (46.2%) | |  |  |  |  |  |  |
| 3. 25.0–29.9 | 117 (25.5%) | |  |  |  |  |  |  |
| 4. >=30.0 | 105 (22.9%) | |  |  |  |  |  |  |
| 5. Missing | 20 (4.4%) | |  |  |  |  |  |  |
| **Tobacco use during pregnancy** |  | |  |  |  |  |  |  |
| 1. No | <430 (<94%) | |  |  |  |  |  |  |
| 2. Yes | 30 (6.5%) | |  |  |  |  |  |  |
| 3. Missing | <5 | |  |  |  |  |  |  |
| **Child Sex** |  | |  |  |  |  |  |  |
| 1. Female | 257 (56.0%) | |  |  |  |  |  |  |
| 2. Male | 202 (44.0%) | |  |  |  |  |  |  |
| **Year of birth** |  | |  |  |  |  |  |  |
| 1. 2006–2010 | 64 (13.9%) | |  |  |  |  |  |  |
| 2. 2011–2015 | 395 (86.1%) | |  |  |  |  |  |  |
| 3. 2016–2020 | 0 (0.0%) | |  |  |  |  |  |  |
| **SRS Second Edition (SRS-2) Version** |  | |  |  |  |  |  |  |
| SRS-2 Preschool (ages 2.5–4.5y) | 310 (67.5%) | |  |  |  |  |  |  |
| SRS-2 School-Age (ages 4–18y) | 149 (32.5%) | |  |  |  |  |  |  |
| Missing |  | |  |  |  |  |  |  |
| **SRS form** |  | |  |  |  |  |  |  |
| Full | 459 (100%) | |  |  |  |  |  |  |
| Short | 0 (0.0%) | |  |  |  |  |  |  |
|  |  | |  |  |  |  |  |  |
|  |  | |  |  |  |  |  |  |
|  |  | |  |  |  |  |  |  |
| **Table S3 (cont)** | **TIDES** | |  |  |  |  |  |  |
| **Clinical ASD diagnosis** |  | |  |  |  |  |  |  |
| 1. No | 430 (93.7%) | |  |  |  |  |  |  |
| 2. Yes | 15 (3.3%) | |  |  |  |  |  |  |
| 3. Missing | 14 (3.1%) | |  |  |  |  |  |  |
| **Total SRS raw score** |  | |  |  |  |  |  |  |
| Mean (SD) | 28.8 ± 18.7 | |  |  |  |  |  |  |
| Median (Q1–Q3) | 25.0 (16.0–35.0) | |  |  |  |  |  |  |
| Min-Max | 0.0–123.0 | |  |  |  |  |  |  |
| **Total SRS T-score** |  | |  |  |  |  |  |  |
| Mean (SD) | 46.3 ± 7.9 | |  |  |  |  |  |  |
| Median (Q1–Q3) | 45.0 (41.0–49.0) | |  |  |  |  |  |  |
| Min-Max | 34.0–85.0 | |  |  |  |  |  |  |
| **Child's age at SRS** |  | |  |  |  |  |  |  |
| Mean (SD) | 5.1 ± 1.8 | |  |  |  |  |  |  |
| Median (Q1–Q3) | 4.0 (4.0–6.0) | |  |  |  |  |  |  |

Abbreviations: ASD, autism spectrum disorder; BMI, body mass index; CANDLE, Conditions Affecting Neurocognitive Development and Learning in Early Childhood; CIOB, Chemicals in Our Bodies; EARLI, Early Autism Risk Longitudinal Investigation; GED, General Educational Development; IKIDS, Illinois Kids Development Study; MADRES, Maternal and Developmental Risks from Environmental and Social

Stressors; MARBLES, University of California - Markers of Autism Risk in Babies – Learning Early Signs; NHBCS, New Hampshire

Birth Cohort Study; NYU CHES, New York University Children’s Health and Environment Study; PETALS, Pregnancy

Environment and Lifestyle Study; SRS, Social Responsiveness Scale; TIDES, The Infant Development and the Environment Study; VCSIP, Vitamin C to Decrease the Effects of Smoking in Pregnancy on Infant Lung Function.

**Table S4.** Overlap between autism diagnosis and SRS T-scores among participants with both clinical history information and SRS assessments.

|  |  | **SRS T-scores** | | | | | | |
| --- | --- | --- | --- | --- | --- | --- | --- | --- |
| **Case status** | **n** | **Mean** | **SD** | **Median** | **25%** | **75%** | **Score**  **60-65** | **Score**  **>=66** |
| **Autism** | 120 | 64.4 | 13.6 | 64 | 53 | 72 | 22 (18.3%) | 51 (42.5%) |
| **No Autism** | 2689 | 48.5 | 7.7 | 47 | 43 | 53 | 131 (4.9%) | 104 (3.9%) |

**Table S5**. Demographic characteristics of participants from 15 ECHO cohorts by study sample inclusion status.

|  | **Included*** | **Not included*** |
| --- | --- | --- |
|  | **N=4,159** | **N=11,001** |
| **Maternal age (years)** |  |  |
| 16–24 | 670 (16.1%) | 1677 (15.2%) |
| 25–29 | 1040 (25.0%) | 2351 (21.4%) |
| 30–34 | 1401 (33.7%) | 3759 (34.2%) |
| 35–47 | 1048 (25.2%) | 3212 (29.2%) |
| Missing | 0 (0%) | 2 |
| **Maternal education** |  |  |
| Up to high school degree, GED, or Equivalent | 1130 (27.3%) | 2577 (27.9%) |
| Some college, no degree | 690 (16.7%) | 2082 (22.5%) |
| Bachelor's degree | 1155 (27.9%) | 2300 (24.9%) |
| Master and above | 1034 (25.0%) | 2285 (24.7%) |
| Missing | 150 | 1757 |
| **Maternal race/ethnicity** |  |  |
| Hispanic All | 543 (13.1%) | 3638 (33.5%) |
| Non-Hispanic Black | 899 (21.7%) | 952 (8.8%) |
| Non-Hispanic Asian | 213 (5.1%) | 806 (7.4%) |
| Non-Hispanic Other (Pacific Islander, American Indian, multi-race and other) | 155 (3.7%) | 386 (3.6%) |
| Non-Hispanic White | 2328 (56.3%) | 5071 (46.7%) |
| Missing/Unknown | 21 | 148 |
|  |  |  |
|  |  |  |
| **Table S5 (cont.)** | **Included*** | **Not included*** |
| **Parity** |  |  |
| Nulliparous | 1743 (42.9%) | 4740 (48.0%) |
| Multiparous | 2321 (57.1%) | 5130 (52.0%) |
| Missing | 95 | 1131 |
| **Pre-pregnancy BMI (kg/m2)** |  |  |
| <18.5 | 123 (3.0%) | 250 (2.7%) |
| 18.5–24.9 | 1869 (46.0%) | 4278 (45.4%) |
| 25.0–29.9 | 1015 (25.0%) | 2581 (27.4%) |
| >=30.0 | 1057 (26.0%) | 2309 (24.5%) |
| Missing | 95 | 1583 |
| **Tobacco use during pregnancy** |  |  |
| No | 3575 (92.8%) | 8491 (95.3%) |
| Yes | 320 (8.3%) | 423 (4.7%) |
| Missing | 264 | 2087 |
| **Child sex** |  |  |
| Female | 2065 (49.7%) | 5317 (48.7%) |
| Male | 2094 (50.3%) | 5590 (51.3%) |
| Missing | 0 | 94 |
| **Year of birth** |  |  |
| 2001–2005 | 0 (0%) | 6 (0.1%) |
| 2006–2010 | 1429 (34.4%) | 890 (8.1%) |
| 2011–2015 | 1900 (45.7%) | 2830 (25.7%) |
| 2016–2020 | 830 (20.0%) | 5640 (51.3%) |
| 2021–2023 | 0 (0%) | 1635 (14.9%) |
|  |  |  |
|  |  |  |
|  |  |  |
| **Table S5 (cont.)** | **Included*** | **Not included*** |
| **Total SRS raw score** |  |  |
| Mean (SD) | 31.3 ± 20.6 | 32.4 ± 20.1 |
| Median (Q1–Q3) | 27.0 (17.0–40.0) | 28.0 (19.0–41.0) |
| Min–Max | 0.0–156.0 | 0.0–151.0 |
| Missing | 871 | 8,714 |
| **Total SRS T-score** |  |  |
| Mean (SD) | 48.8 ± 8.6 | 49.4 ± 8.2 |
| Median (Q1–Q3) | 47.0 (43.0–53.0) | 48.0 (44.0–53.0) |
| Min–Max | 34.0–99.0 | 34.0–97.0 |
| Missing | 871 | 8,714 |
| **SRS subscore in Social Communication and Interaction T-score** |  |  |
| Mean (SD) | 48.1 ± 8.5 | 49.0 ± 8.3 |
| Median (Q1-Q3) | 46.0 (42.0–51.0) | 47.0 (43.0–53.0) |
| Min–Max | 35.0–100.0 | 35.0–93.0 |
| Missing | 1411 | 8,702 |
| **SRS subscale in Restricted Interests and Repetitive Behaviors T-score** |  |  |
| Mean (SD) | 48.7 ± 8.5 | 48.9 ± 8.3 |
| Median (Q1–Q3) | 46.0 (43.0–52.0) | 46.0 (44.0–52.0) |
| Min–Max | 40.0–104.0 | 40.0–104.0 |
| Missing | 1411 | 8,702 |
|  |  |  |
|  |  |  |
| **Table S5 (cont.)** | **Included*** | **Not included*** |
| **SRS Second Edition (SRS-2) Version** |  |  |
| SRS-2 Preschool (ages 2.5–4.5y) | 961 (29.2%) | 591 (5.4%) |
| SRS-2 School-Age (ages 4–18y) | 2327 (70.8%) | 1710 (15.5%) |
| Missing | 871 | 8700 (79.1%) |
| **Child's age at SRS (years)** |  |  |
| Mean (SD) | 6.9 ± 3.3 | 5.4 ± 2.3 |
| Median (Q1–Q3) | 6.0 (4.0–10.0) | 4.0 (4.0–7.0) |
| Min–Max | 2.9–16 | 2.6–18.0 |
| Missing | 871 | 9,045 |
| **Clinical ASD diagnosis** |  |  |
| No | 3501 (95.1%) | 2347 (95.5%) |
| Yes | 179 (4.9%) | 111 (4.5%) |
| Missing | 479 | 8543 (77.7%) |
| **Child's age at last medical history follow-up** |  |  |
| Mean (SD) | 6.8 (2.8) | 4.7 (1.5) |
| Median (Q1–Q3) | 6.2 (4.6–9.4) | 4.5 (3.6–6.5) |
| Min–Max | 1.1–13.0 | 3.6–6.5 |
| Missing | 528 | 9,002 |

*Percentages were calculated missing observations removed from denominator.

Abbreviations: ASD, autism spectrum disorder; BMI, body mass index; GED, General Educational Development; SRS, Social Responsiveness Scale

**Table S6**. Distributions of OPEs (dilution corrected, untransformed values, μg/L) and % below the limit of detection (LOD) in A) pooled sample, B) SRS Sample, and C) Autism Diagnosis sample.

**A**

| **Analyte** | **n** | **LOD** | **N<LOD** | **% above LOD** | **5th Percentile** | **25th Percentile** | **50th Percentile** | **75th Percentile** | **95th Percentile** |
| --- | --- | --- | --- | --- | --- | --- | --- | --- | --- |
| DBUP/DIBP | 4159 | 0.04 | 187 | 95.5 | 0.07 | 0.13 | 0.19 | 0.3 | 0.84 |
| DPHP | 4159 | 0.03 | 21 | 99.5 | 0.27 | 0.56 | 0.95 | 1.83 | 9.03 |
| BDCPP | 4159 | 0.02 | 532 | 87.2 | <LOD | 0.33 | 0.88 | 1.75 | 5.14 |
| BCPP | 4159 | 0.02 | 2000 | 51.9 | <LOD | <LOD | 0.09 | 0.75 | 3.6 |
| BCETP | 4159 | 0.02 | 1214 | 70.8 | <LOD | <LOD | 0.56 | 1.69 | 8.6 |
| BBOEP | 4159 | 0.02 | 1385 | 66.7 | <LOD | <LOD | 0.05 | 0.09 | 0.25 |
| BMPP | 4159 | 0.01 | 2653 | 36.2 | <LOD | <LOD | <LOD | 0.03 | 0.13 |
| BEHP | 4159 | 0.02 | 2932 | 29.5 | <LOD | <LOD | <LOD | 0.04 | 0.57 |
| DPRP | 4159 | 0.03 | 3069 | 26.2 | <LOD | <LOD | <LOD | 0.04 | 0.34 |
|  |  |  |  |  |  |  |  |  |  |
| **B** |  |  |  |  |  |  |  |  |  |
| **Analyte** | **n** | **LOD** | **N<LOD** | **% above LOD** | **5th Percentile** | **25th Percentile** | **50th Percentile** | **75th Percentile** | **95th Percentile** |
| DBUP/DIBP | 3288 | 0.04 | 161 | 95.1 | 0.07 | 0.12 | 0.19 | 0.3 | 0.79 |
| DPHP | 3288 | 0.03 | 16 | 99.5 | 0.27 | 0.54 | 0.93 | 1.84 | 10.43 |
| BDCPP | 3288 | 0.02 | 424 | 87.1 | <LOD | 0.33 | 0.85 | 1.67 | 5.29 |
| BCPP | 3288 | 0.02 | 1578 | 52 | <LOD | <LOD | 0.1 | 0.74 | 3.53 |
| BCETP | 3288 | 0.02 | 986 | 70 | <LOD | <LOD | 0.53 | 1.66 | 8.22 |
| BBOEP | 3288 | 0.02 | 1108 | 66.3 | <LOD | <LOD | 0.05 | 0.09 | 0.26 |
| BMPP | 3288 | 0.01 | 2111 | 35.8 | <LOD | <LOD | <LOD | 0.03 | 0.13 |
| BEHP | 3288 | 0.02 | 2328 | 29.2 | <LOD | <LOD | <LOD | 0.04 | 0.59 |
| DPRP | 3288 | 0.03 | 2456 | 25.3 | <LOD | <LOD | <LOD | 0.03 | 0.35 |
|  |  |  |  |  |  |  |  |  |  |
|  |  |  |  |  |  |  |  |  |  |
| **Table S6 (Cont.)** |  |  |  |  |  |  |  |  |  |
| **C** |  |  |  |  |  |  |  |  |  |
| **Analyte** | **n** | **LOD** | **N<LOD** | **% above LOD** | **5th Percentile** | **25th Percentile** | **50th Percentile** | **75th Percentile** | **95th Percentile** |
| DBUP/DIBP | 3680 | 0.04 | 158 | 95.7 | 0.07 | 0.13 | 0.19 | 0.3 | 0.82 |
| DPHP | 3680 | 0.03 | 18 | 99.5 | 0.29 | 0.59 | 0.98 | 1.88 | 9.24 |
| BDCPP | 3680 | 0.02 | 467 | 87.3 | <LOD | 0.35 | 0.92 | 1.84 | 5.37 |
| BCPP | 3680 | 0.02 | 1770 | 51.9 | <LOD | <LOD | 0.1 | 0.76 | 3.55 |
| BCETP | 3680 | 0.02 | 1030 | 72 | <LOD | <LOD | 0.63 | 1.81 | 9.22 |
| BBOEP | 3680 | 0.02 | 1248 | 66.1 | <LOD | <LOD | 0.05 | 0.09 | 0.26 |
| BMPP | 3680 | 0.01 | 2348 | 36.2 | <LOD | <LOD | <LOD | 0.03 | 0.13 |
| BEHP | 3680 | 0.02 | 2598 | 29.4 | <LOD | <LOD | <LOD | 0.04 | 0.5 |
| DPRP | 3680 | 0.04 | 2727 | 25.9 | <LOD | <LOD | <LOD | 0.03 | 0.34 |

Abbreviations: BBOEP, bis(butoxyethyl) phosphate; BCETP, bis(2-chloroethyl) phosphate; BCPP, bis(1-chloro-2-propyl) phosphate; BDCPP, bis(1,3-dichloro-2-propyl) phosphate; BEHP, bis(2-ethylhexyl) phosphate; BMPP, bis(2-methylphenyl) phosphate; DBUP/DIBP, composite of dibutyl phosphate and di-isobutyl phosphate; DPHP, di(2-propylheptyl) phthalate; DPRP, dipropyl phosphate; LOD, limit of detection; OPE, organophosphate ester flame retardant and plasticizer.

**Table S7.** Associations of OPEs and SRS raw scores in linear models fitted with generalized estimating equations with clustering by ECHO cohort.

|  |  | **SRS raw scores** | |
| --- | --- | --- | --- |
| **OPE analyte** | **Level** | **β_crude_ (95% CI) ^a^ n=3288** | **β_adj_ (95% CI) ^b^ n=3288** |
| **Continuous** |  |  |  |
| DBUP/DIBP | Continuous | -0.01 (-0.70, 0.67) | 0.07 (-0.56, 0.70) |
| DPHP | Continuous | 0.37 (-0.19, 0.93) | -0.08 (-0.29, 0.13) |
| BDCPP | Continuous | 0.12 (-0.05, 0.30) | 0.06 (-0.09, 0.21) |
|  |  |  |  |
| **Categorical** |  |  |  |
| BCPP | > Median | -0.11 (-2.23, 2.00) | 1.14 (-0.78, 3.07) |
|  | <= Median | 1.01 (-0.52, 2.55) | 2.07 (0.90, 3.24) |
|  | <LOD | ref | ref |
| BCETP | > Median | 0.87 (-0.62, 2.37) | 0.19 (-1.26, 1.63) |
|  | <= Median | 0.20 (-1.23, 1.63) | 0.61 (-0.72, 1.94) |
|  | <LOD | ref | ref |
| BBOEP | > Median | 3.70 (1.80, 5.60) | 2.72 (1.25, 4.19) |
|  | <= Median | 2.35 (-0.86, 5.56) | 1.30 (-1.49, 4.09) |
|  | <LOD | ref | ref |
| **Detect/non-detect** |  |  |  |
| BMPP | >=LOD | 0.87 (-2.02, 3.75) | 0.30 (-1.59, 2.19) |
|  | <LOD | ref | ref |
| BEHP | >=LOD | 0.25 (-1.22, 1.72) | 0.22 (-1.06, 1.50) |
|  | <LOD | ref | ref |
| DPRP | >=LOD | -0.82 (-2.49, 0.85) | -0.42 (-1.95, 1.11) |
|  | <LOD | ref | ref |

^a^Adjusted for cohort.

^b^Adjusted MICE models included ECHO cohort, maternal age (continuous), child age at SRS (continuous), race/ethnicity, pre-pregnancy BMI, educational attainment, sex, parity, and smoking during pregnancy. T-score is standardized for SRS version (preschool, school-age) and child’s sex.

Abbreviations: BBOEP, bis(butoxyethyl) phosphate; BCPP, bis(1-chloro-2-propyl) phosphate; BCETP, bis(2-chloroethyl) phosphate; BDCPP, bis(1,3-dichloro-2-propyl) phosphate; BEHP, bis(2-ethylhexyl) phosphate; BMPP, bis(2-methylphenyl) phosphate; BMI, body mass index; CI, confidence interval; DBUP/DIBP, composite of dibutyl phosphate and di-isobutyl phosphate; DPHP, di(2-propylheptyl) phthalate; DPRP, dipropyl phosphate; ECHO, Environmental influences on Child Health Outcomes; LOD, limit of detection; MICE, multiple imputation by chained equations; OPE, organophosphate ester flame retardant and plasticizer; SRS, Social Responsiveness Scale.

**Table S8**. Associations of OPEs and T-scores on SRS subscales in linear models fitted with generalized estimating equations with clustering by ECHO cohort.

|  |  | **SRS Social Communication and Interaction subscale (T-score)** | **SRS Restricted Interests and Repetitive Behavior subscale (T-score)** |
| --- | --- | --- | --- |
| **OPE analyte** | **Level** | **β_adj_ (95% CI) ^a^ n=2748** | **β_adj_ (95% CI) ^a^ n=2748** |
| **Continuous** |  |  |  |
| DBUP/DIBP | Continuous | -0.00 (-0.30, 0.29) | 0.01 (-0.26, 0.29) |
| DPHP | Continuous | -0.07 (-0.18, 0.04) | -0.06 (-0.18, 0.06) |
| BDCPP | Continuous | -0.01 (-0.11, 0.09) | 0.04 (-0.03, 0.11) |
|  |  |  |  |
| **Categorical** |  |  |  |
| BCPP | > Median | 0.49 (-0.28, 1.26) | 0.09 (-0.55, 0.74) |
|  | <= Median | 1.14 (0.47, 1.81) | 0.69 (0.04, 1.33) |
|  | <LOD |  |  |
| BCETP | > Median | 0.18 (-0.37, 0.73) | 0.25 (-0.42, 0.93) |
|  | <= Median | 0.34 (-0.33, 1.02) | 0.40 (-0.28, 1.08) |
|  | <LOD |  |  |
| BBOEP | > Median | 0.75 (0.15, 1.35) | 0.65 (0.07, 1.22) |
|  | <= Median | 0.67 (-0.63, 1.96) | 0.24 (-1.03, 1.51) |
|  | <LOD |  |  |
| **Detect/non-detect** |  |  |  |
| BMPP | >=LOD | 0.10 (-0.53, 0.74) | -0.39 (-0.96, 0.19) |
|  | <LOD |  |  |
| BEHP | >=LOD | 0.31 (-0.21, 0.82) | 0.53 (0.04, 1.03) |
|  | <LOD |  |  |
| DPRP | >=LOD | -0.47 (-0.95, 0.01) | -0.45 (-1.30, 0.41) |
|  | <LOD |  |  |

^a^Adjusted MICE models included ECHO cohort, maternal age (continuous), child age at SRS (continuous), race/ethnicity, pre-pregnancy BMI, educational attainment, sex, parity, and smoking during pregnancy. T-score is standardized for SRS version (preschool, school-age) and child’s sex.

Abbreviations: BBOEP, bis(butoxyethyl) phosphate; BCPP, bis(1-chloro-2-propyl) phosphate; BCETP, bis(2-chloroethyl) phosphate; BDCPP, bis(1,3-dichloro-2-propyl) phosphate; BEHP, bis(2-ethylhexyl) phosphate; BMPP, bis(2-methylphenyl) phosphate; BMI, body mass index; CI, confidence interval; DBUP/DIBP, composite of dibutyl phosphate and di-isobutyl phosphate; DPHP, di(2-propylheptyl) phthalate; DPRP, dipropyl phosphate; ECHO, Environmental influences on Child Health Outcomes; LOD, limit of detection; MICE, multiple imputation by chained equations; OPE, organophosphate ester flame retardant and plasticizer; SRS, Social Responsiveness Scale.

**Table S9.** Associations of OPEs and SRS clinical cutpoints in logistic models fitted with generalized estimating equations with clustering by ECHO cohort.

|  |  | **SRS cutpoints (60-65^a^ vs. <60) (Mild vs No autistic traits)** | **SRS cutpoints (≥66^b^ vs. <60) Moderate/Severe vs No autistic traits** |
| --- | --- | --- | --- |
| **OPE analyte** | **Level** | **OR_adj_ (95% CI) ^c^ n=3166** | **OR_adj_ (95% CI) ^c^ n=3121** |
| **Continuous** |  |  |  |
| DBUP/DIBP | Continuous | 1.09 (0.92, 1.29) | 1.01 (0.92, 1.11) |
| DPHP | Continuous | 1.03 (0.98, 1.08) | 1.01 (0.97, 1.06) |
| BDCPP | Continuous | 1.02 (0.97, 1.08) | 1.01 (0.98, 1.04) |
|  |  |  |  |
| **Categorical** |  |  |  |
| BCPP | > Median | 1.08 (0.68, 1.73) | 1.15 (0.80, 1.67) |
|  | <= Median | 1.13 (0.73, 1.75) | 1.28 (1.02, 1.60) |
|  | <LOD |  |  |
| BCETP | > Median | 1.43 (1.07, 1.91) | 0.88 (0.67, 1.17) |
|  | <= Median | 0.93 (0.64, 1.35) | 1.01 (0.82, 1.23) |
|  | <LOD |  |  |
| BBOEP | > Median | 1.30 (0.87, 1.95) | 1.38 (1.11, 1.71) |
|  | <= Median | 1.21 (0.82, 1.78) | 1.42 (1.05, 1.91) |
|  | <LOD |  |  |
| **Detect/non-detect** | |  |  |
| BMPP | >=LOD | 0.91 (0.70, 1.18) | 1.08 (0.90, 1.30) |
|  | <LOD |  |  |
| BEHP | >=LOD | 1.19 (0.88, 1.63) | 1.04 (0.83, 1.32) |
|  | <LOD |  |  |
| DPRP | >=LOD | 1.08 (0.75, 1.54) | 1.05 (0.83, 1.34) |
|  | <LOD |  |  |

^a^ n=167 children with SRS T scores 60-65

^b^ n=172 children with SRS T scores >=66

^c^ Adjusted MICE models included ECHO cohort, maternal age (continuous), child age at SRS (continuous), race/ethnicity, pre-pregnancy BMI, educational attainment, sex, parity, and smoking during pregnancy. T-score is standardized for SRS version (preschool, school-age) and child’s sex.

Abbreviations: BBOEP, bis(butoxyethyl) phosphate; BCPP, bis(1-chloro-2-propyl) phosphate; BCETP, bis(2-chloroethyl) phosphate; BDCPP, bis(1,3-dichloro-2-propyl) phosphate; BEHP, bis(2-ethylhexyl) phosphate; BMPP, bis(2-methylphenyl) phosphate; BMI, body mass index; CI, confidence interval; DBUP/DIBP, composite of dibutyl phosphate and di-isobutyl phosphate; DPHP, di(2-propylheptyl) phthalate; DPRP, dipropyl phosphate; ECHO, Environmental influences on Child Health Outcomes; LOD, limit of detection; MICE, multiple imputation by chained equations; OPE, organophosphate ester flame retardant and plasticizer; SRS, Social Responsiveness Scale.

**Table S10**. Birth year adjustment sensitivity analysis of associations of OPEs and SRS T-scores and Clinical autism diagnosis in models fitted with generalized estimating equations.

|  |  | **SRS (T-score)** |  | **Clinical Autism Diagnosis** |  |
| --- | --- | --- | --- | --- | --- |
| **OPE analyte** | **Level** | **β_adj_ (95% CI) ^a^ n=2748** | **p-value^b^** | **OR_adj_ (95% CI) ^a^  n=3,680** | **p-value^b^** |
| **Continuous/Tertiles** |  |  |  |  |  |
| DBUP/DIBP | Continuous | 0.02 (-0.26, 0.31) |  | 1.02 (0.95, 1.10) |  |
|  | Tertile 3 | -0.20 (-0.96, 0.55) | 0.61 | 0.93 (0.73, 1.18) | 0.58 |
|  | Tertile 2 | 0.10 (-0.36, 0.56) |  | 1.08 (0.76, 1.54) |  |
|  | Tertile 1 | ref |  | ref |  |
| DPHP | Continuous | 0.01 (-0.10, 0.11) |  | 0.93 (0.84, 1.02) |  |
|  | Tertile 3 | 0.00 (-0.54, 0.54) | 0.37 | 0.78 (0.48, 1.26) | 0.32 |
|  | Tertile 2 | 0.25 (-0.20, 0.70) |  | 0.95 (0.71, 1.27) |  |
|  | Tertile 1 |  |  | ref |  |
| BDCPP | Continuous | 0.02 (-0.05, 0.09) |  | 1.00 (0.99, 1.02) |  |
|  | Tertile 3 | 0.12 (-0.43, 0.67) | 0.17 | 0.94 (0.79, 1.13) | 0.49 |
|  | Tertile 2 | 0.45 (-0.03, 0.93) |  | 1.03 (0.79, 1.33) |  |
|  | Tertile 1 | ref |  | ref |  |
| **Categorical** |  |  |  |  |  |
| BCPP | > Median^c^ | 0.29 (-0.48, 1.06) | 0.014 | 0.87 (0.57, 1.33) | 0.14 |
|  | <= Median | 0.70 (0.23, 1.17) |  | 1.18 (0.87, 1.60) |  |
|  | <LOD | ref |  | ref |  |
| BCETP | > Median^c^ | 0.04 (-0.47, 0.56) | 0.90 | 0.75 (0.60, 0.94) | 0.043 |
|  | <= Median | 0.09 (-0.47, 0.66) |  | 0.83 (0.64, 1.07) |  |
|  | <LOD | ref |  | ref |  |
| BBOEP | > Median^c^ | 0.92 (0.31, 1.54) | 0.001 | 1.26 (1.11, 1.44) | <0.001 |
|  | <= Median | 0.45 (-0.67, 1.56) |  | 0.94 (0.76, 1.17) |  |
|  | <LOD | ref |  | ref |  |
| **Detect/non-detect** |  |  |  |  |  |
| BMPP | >=LOD | -0.02 (-0.74, 0.70) |  | 1.34 (0.98, 1.84) |  |
|  | <LOD | ref |  | ref |  |
| BEHP | >=LOD | 0.16 (-0.38, 0.69) |  | 1.15 (0.83, 1.58) |  |
|  | <LOD | ref |  | ref |  |
| DPRP | >=LOD | -0.22 (-0.83, 0.39) |  | 0.87 (0.60, 1.27) |  |
|  | <LOD | ref |  | ref |  |

^a^Adjusted MICE models included ECHO cohort, maternal age (continuous), race/ethnicity, pre-pregnancy BMI, educational attainment, sex, parity, smoking during pregnancy, and birth year (continuous). The SRS T-score model is additionally adjusted for child age at SRS.

^b^ Overall p-value of the three-category OPE exposure variable calculated from a Type III Wald Test.

^c^ Median of the detectable values

Abbreviations: BBOEP, bis(butoxyethyl) phosphate; BCPP, bis(1-chloro-2-propyl) phosphate; BCETP, bis(2-chloroethyl) phosphate; BDCPP, bis(1,3-dichloro-2-propyl) phosphate; BEHP, bis(2-ethylhexyl) phosphate; BMPP, bis(2-methylphenyl) phosphate; BMI, body mass index; CI, confidence interval; DBUP/DIBP, composite of dibutyl phosphate and di-isobutyl phosphate; DPHP, di(2-propylheptyl) phthalate; DPRP, dipropyl phosphate; ECHO, Environmental influences on Child Health Outcomes; LOD, limit of detection; MICE, multiple imputation by chained equations; OPE, organophosphate ester flame retardant and plasticizer; SRS, Social Responsiveness Scale.

**Table S11**. Associations between OPEs and SRS total T-score in linear models fitted with generalized estimating equations with clustering by ECHO cohort, stratified by SRS-2 form completed

|  | Pre-school form ^a^ | | School-age form ^b^ |
| --- | --- | --- | --- |
|  | **SRS total T-score** | |  |
| **OPE analyte** | **Level** | **β_adj_ (95% CI) ^c^ n=961** | **β_adj_ (95% CI) ^c^ n=2327** |
| **Continuous** |  |  |  |
| DBUP/DIBP | Continuous | 0.11 (-0.32, 0.54) | 0.00 (-0.24, 0.25) |
| DPHP | Continuous | -0.12 (-0.27, 0.03) | -0.00 (-0.10, 0.09) |
| BDCPP | Continuous | 0.09 (-0.01, 0.19) | 0.00 (-0.08, 0.08) |
|  |  |  |  |
| **Categorical** |  |  |  |
| BCPP | > Median | -0.31 (-0.77, 0.14) | 0.83 (-0.02, 1.68) |
|  | <= Median | 0.90 (0.10, 1.69) | 0.73 (0.21, 1.25) |
|  | <LOD |  |  |
| BCETP | > Median | 0.40 (-0.34, 1.15) | -0.01 (-0.51, 0.49) |
|  | <= Median | 0.16 (-0.76, 1.08) | 0.26 (-0.55, 1.07) |
|  | <LOD |  |  |
| BBOEP | > Median | 1.04 (0.33, 1.75) | 1.11 (0.27, 1.96) |
|  | <= Median | 0.36 (-0.33, 1.05) | 0.57 (-0.88, 2.01) |
|  | <LOD |  |  |
| **Detect/non-detect** |  |  |  |
| BMPP | >=LOD | -0.53 (-1.06, 0.00) | 0.36 (-0.53, 1.24) |
|  | <LOD |  |  |
| BEHP | >=LOD | -0.28 (-1.02, 0.45) | 0.23 (-0.58, 1.04) |
|  | <LOD |  |  |
| DPRP | >=LOD | -0.68 (-1.27, -0.09) | 0.05 (-0.68, 0.78) |
|  | <LOD |  |  |

^a^ Restricted to participants who completed the pre-school version of the SRS (participants were 2.5–5 years old)

^b^Restricted to participants who completed the child version of SRS (participants were 4–16 years old)

^c^Adjusted MICE models included ECHO cohort, maternal age (continuous), child age at SRS (continuous), race/ethnicity, pre-pregnancy BMI, educational attainment, sex, parity, and smoking during pregnancy. T-score is standardized for SRS version (preschool, school-age) and child’s sex.

Abbreviations: BBOEP, bis(butoxyethyl) phosphate; BCPP, bis(1-chloro-2-propyl) phosphate; BCETP, bis(2-chloroethyl) phosphate; BDCPP, bis(1,3-dichloro-2-propyl) phosphate; BEHP, bis(2-ethylhexyl) phosphate; BMPP, bis(2-methylphenyl) phosphate; BMI, body mass index; CI, confidence interval; DBUP/DIBP, composite of dibutyl phosphate and di-isobutyl phosphate; DPHP, di(2-propylheptyl) phthalate; DPRP, dipropyl phosphate; ECHO, Environmental influences on Child Health Outcomes; LOD, limit of detection; MICE, multiple imputation by chained equations; OPE, organophosphate ester flame retardant and plasticizer; SRS, Social Responsiveness Scale.

**Table S12**. Associations of OPEs and SRS T-score in linear models fitted with generalized estimating equations with clustering by ECHO cohort, after excluding MARBLES and EARLI

|  |  | **SRS Total T-Score** |
| --- | --- | --- |
| **OPE analyte** | **Level** | **β_adj_ (95% CI) ^a^ n=2948** |
| **Continuous** |  |  |
| DBUP/DIBP | Continuous | -0.04 (-0.27, 0.19) |
| DPHP | Continuous | 0.01 (-0.09, 0.10) |
| BDCPP | Continuous | 0.00 (-0.07, 0.07) |
|  |  |  |
| **Categorical** |  |  |
| BCPP | > Median | 0.39 (-0.43, 1.21) |
|  | <= Median | 0.56 (0.12, 1.01) |
|  | <LOD |  |
| BCETP | > Median | 0.10 (-0.40, 0.60) |
|  | <= Median | 0.37 (0.02, 0.72) |
|  | <LOD |  |
| BBOEP | > Median | 1.06 (0.46, 1.65) |
|  | <= Median | 0.50 (-0.63, 1.63) |
|  | <LOD |  |
| **Detect/non-detect** |  |  |
| BMPP | >=LOD | -0.02 (-0.80, 0.77) |
|  | <LOD |  |
| BEHP | >=LOD | 0.07 (-0.42, 0.55) |
|  | <LOD |  |
| DPRP | >=LOD | -0.19 (-0.81, 0.43) |
|  | <LOD |  |

^a^Adjusted MICE models included ECHO cohort, maternal age (continuous), child age at SRS (continuous), race/ethnicity, pre-pregnancy BMI, educational attainment, sex, parity, and smoking during pregnancy. T-score is standardized for SRS version (preschool, school-age) and child’s sex.

Abbreviations: BBOEP, bis(butoxyethyl) phosphate; BCPP, bis(1-chloro-2-propyl) phosphate; BCETP, bis(2-chloroethyl) phosphate; BDCPP, bis(1,3-dichloro-2-propyl) phosphate; BEHP, bis(2-ethylhexyl) phosphate; BMPP, bis(2-methylphenyl) phosphate; BMI, body mass index; CI, confidence interval; DBUP/DIBP, composite of dibutyl phosphate and di-isobutyl phosphate; DPHP, di(2-propylheptyl) phthalate; DPRP, dipropyl phosphate; ECHO, Environmental influences on Child Health Outcomes; LOD, limit of detection; MICE, multiple imputation by chained equations; OPE, organophosphate ester flame retardant and plasticizer; SRS, Social Responsiveness Scale.

**Table S13.** Associations of OPEs and ASD diagnoses in logistic models fitted with generalized estimating equations with clustering by ECHO cohort, after excluding MARBLES and EARLI

|  |  | **Clinical Autism Diagnosis** |
| --- | --- | --- |
| **OPE analyte** | **Level** | **OR_adj_ (95% CI) ^a^ n=3,341** |
| **Continuous** |  |  |
| DBUP/DIBP | Continuous | 1.01 (0.85, 1.19) |
| DPHP | Continuous | 0.92 (0.82, 1.02) |
| BDCPP | Continuous | 1.01 (0.97, 1.04) |
|  |  |  |
| **Categorical** |  |  |
| BCPP | > Median | 0.99 (0.49, 2.03) |
|  | <= Median | 1.01 (0.49, 2.05) |
|  | <LOD |  |
| BCETP | > Median | 0.69 (0.43, 1.10) |
|  | <= Median | 0.76 (0.45, 1.28) |
|  | <LOD |  |
| BBOEP | > Median | 1.41 (1.08, 1.85) |
|  | <= Median | 1.06 (0.72, 1.57) |
|  | <LOD |  |
| **Detect/non-detect** |  |  |
| BMPP | >=LOD | 1.66 (0.98, 2.81) |
|  | <LOD |  |
| BEHP | >=LOD | 1.55 (0.87, 2.78) |
|  | <LOD |  |
| DPRP | >=LOD | 1.25 (0.86, 1.83) |
|  | <LOD |  |

^a^Adjusted MICE models included ECHO cohort, maternal age (continuous), race/ethnicity, pre-pregnancy BMI, educational attainment, sex, parity, and smoking during pregnancy.

Abbreviations: BBOEP, bis(butoxyethyl) phosphate; BCPP, bis(1-chloro-2-propyl) phosphate; BCETP, bis(2-chloroethyl) phosphate; BDCPP, bis(1,3-dichloro-2-propyl) phosphate; BEHP, bis(2-ethylhexyl) phosphate; BMPP, bis(2-methylphenyl) phosphate; BMI, body mass index; CI, confidence interval; DBUP/DIBP, composite of dibutyl phosphate and di-isobutyl phosphate; DPHP, di(2-propylheptyl) phthalate; DPRP, dipropyl phosphate; EARLI, Early Autism Risk Longitudinal Investigation; ECHO, Environmental influences on Child Health Outcomes; LOD, limit of detection; MARBLES, University of California - Markers of Autism Risk in Babies – Learning Early Signs; MICE, multiple imputation by chained equations; OPE, organophosphate ester flame retardant and plasticizer; SRS, Social Responsiveness Scale.

**Table S14.** Associations of OPEs and autism diagnoses in logistic models fitted with generalized estimating equations, restricted to 4 cohorts with >= 15 autism cases: Marbles, EARLI, Candle, and TIDES.

|  |  | **Clinical Autism Diagnosis** |
| --- | --- | --- |
| **OPE analyte** | **Level** | **OR_adj_ (95% CI) ^a^ n=1,980** |
| **Continuous** |  |  |
| DBUP/DIBP | Continuous | 1.01 (0.97, 1.06) |
| DPHP | Continuous | 0.92 (0.85, 1.00) |
| BDCPP | Continuous | 1.00 (1.00, 1.01) |
|  |  |  |
| **Categorical** |  |  |
| BCPP | > Median | 0.92 (0.64, 1.34) |
|  | <= Median | 1.22 (0.97, 1.52) |
|  | <LOD | ref |
| BCETP | > Median | 0.86 (0.75, 0.98) |
|  | <= Median | 0.92 (0.74, 1.15) |
|  | <LOD | ref |
| BBOEP | > Median | 1.17 (1.07,1.27) |
|  | <= Median | 0.89 (0.72, 1.10) |
|  | <LOD | ref |
| **Detect/non-detect** |  |  |
| BMPP | >=LOD | 1.26 (0.95, 1.66) |
|  | <LOD | ref |
| BEHP | >=LOD | 0.92 (0.80, 1.05) |
|  | <LOD | ref |
| DPRP | >=LOD | 0.83 (0.60, 1.14) |
|  | <LOD | ref |

^a^Adjusted MICE models included ECHO cohort, maternal age (continuous), race/ethnicity, pre-pregnancy BMI, educational attainment, sex, parity, and smoking during pregnancy.

Abbreviations: BBOEP, bis(butoxyethyl) phosphate; BCPP, bis(1-chloro-2-propyl) phosphate; BCETP, bis(2-chloroethyl) phosphate; BDCPP, bis(1,3-dichloro-2-propyl) phosphate; BEHP, bis(2-ethylhexyl) phosphate; BMPP, bis(2-methylphenyl) phosphate; BMI, body mass index; CI, confidence interval; DBUP/DIBP, composite of dibutyl phosphate and di-isobutyl phosphate; DPHP, di(2-propylheptyl) phthalate; DPRP, dipropyl phosphate; ECHO, Environmental influences on Child Health Outcomes; LOD, limit of detection; MICE, multiple imputation by chained equations; OPE, organophosphate ester flame retardant and plasticizer; SRS, Social Responsiveness Scale.

**S Figure 1:** Flow chart of included participants

**N=7,038** Unique Pregnancies with Prenatal OPE Measurements from the HHEAR lab and Measures of Urinary Dilution

**N=145 pregnancies**
n=10 twins/triplets
n=64 with missing singleton status
n=71 pregnancies with no ECHO registered child

**N=6,893** ECHO singleton children

**SRS outcome**

**N=6,893** ECHO singleton children

**ASD outcome**

**N=6,893** ECHO singleton children

**n=3,162** children without ASD diagnosis information

**n=3,466** children no second edition SRS form

**n=2** children total SRS T-score missing

**N=3,731** ECHO singleton children have ASD diagnosis information from medical history forms

**N=3,425** ECHO singleton children have second edition SRS form with total SRS T-score calculated. N=4,700 forms

**n=37** younger siblings from the same family were excluded

**n=14** children from Atlanta ECHO Cohort of Emory University

**n=75** children age at SRS <2.5 or missing for preschool SRS forms. **N=117** preschool SRS forms excluded

**N=3,680** ECHO singleton children after keeping older sibling for each family unit and keeping cohort with greater than or equal to 30 children

**N=3,350** ECHO singleton children after restricting age at SRS >=2.5. Preschool version age range 2.85-5.0; School version age range 4-18. N=4,583 forms

**n=58** younger sibling from the same family were excluded

**n=4** children from Atlanta ECHO Cohort of Emory University

**N=3,288** ECHO singleton children after keeping older sibling for each family unit and keeping cohort with greater than or equal to 15 children. n=3,288 forms after keeping most recent form for each child

**Figure S2**. Direct acyclic graph (made with Daggity^a^) of the relationship between pregnancy urine concentrations of organophosphate ester (OPE) exposure and autism-related outcomes

^a^Johannes Textor, Benito van der Zander, Mark K. Gilthorpe, Maciej Liskiewicz, George T.H. Ellison. Robust causal inference using directed acyclic graphs: the R package 'dagitty'. International Journal of Epidemiology 45(6):1887-1894, 2016.

Abbreviations: ASD, autism spectrum disorder; Gest week, gestational week; OPE, organophosphate ester flame retardant and plasticizer; SES, socioeconomic status.

**Figure S3**. Boxplots of OPE analytes (log2-transformed, dilution corrected) in the pooled ECHO sample

Abbreviations: BBOEP, bis(butoxyethyl) phosphate; BCPP, bis(1-chloro-2-propyl) phosphate; BCETP, bis(2-chloroethyl) phosphate; BDCPP, bis(1,3-dichloro-2-propyl) phosphate; BEHP, bis(2-ethylhexyl) phosphate; BMPP, bis(2-methylphenyl) phosphate; DBUP/DIBP, composite of dibutyl phosphate and di-isobutyl phosphate; DPHP, di(2-propylheptyl) phthalate; DPRP, dipropyl phosphate; ECHO, Environmental influences on Child Health Outcomes; OPE, organophosphate ester flame retardant and plasticizer.

**Figure S4**. Boxplots of OPE analyte distributions (log2-transformed, dilution corrected) by ECHO cohort

**Figure S4 (Cont)**. Boxplots of OPE analyte distributions (log2-transformed, dilution corrected) by ECHO cohort

**Figure S4 (Cont)**. Boxplots of OPE analyte distributions (log2-transformed, dilution corrected) by ECHO cohort

Abbreviations: BBOEP, bis(butoxyethyl) phosphate; BCPP, bis(1-chloro-2-propyl) phosphate; BCETP, bis(2-chloroethyl) phosphate; BDCPP, bis(1,3-dichloro-2-propyl) phosphate; BEHP, bis(2-ethylhexyl) phosphate; BMPP, bis(2-methylphenyl) phosphate; CANDLE, Conditions Affecting Neurocognitive Development and Learning in Early Childhood; CIOB, Chemicals in Our Bodies; DBUP/DIBP, composite of dibutyl phosphate and di-isobutyl phosphate; DPHP, di(2-propylheptyl) phthalate; DPRP, dipropyl phosphate; EARLI, Early Autism Risk Longitudinal Investigation; ECHO, Environmental influences on Child Health Outcomes; IKIDS, Illinois Kids Development Study; MADRES, Maternal and Developmental Risks from Environmental and Social Stressors; MARBLES, University of California - Markers of Autism Risk in Babies – Learning Early Signs; NHBCS, New Hampshire Birth Cohort Study; NYU CHES, New York University Children’s Health and Environment Study; OPE, organophosphate ester flame retardant and plasticizer; PETALS, Pregnancy Environment and Lifestyle Study; TIDES, The Infant Development and the Environment Study; VCSIP, Vitamin C to Decrease the Effects of Smoking in Pregnancy on Infant Lung Function.

**Figure S5**. Spearman correlations on all OPEs, in the full sample, and by SRS or autism subsample, imputed, dilution corrected.

Abbreviations: ASD, autism spectrum disorder; BBOEP, bis(butoxyethyl) phosphate; BCPP, bis(1-chloro-2-propyl) phosphate; BCETP, bis(2-chloroethyl) phosphate; BDCPP, bis(1,3-dichloro-2-propyl) phosphate; BEHP, bis(2-ethylhexyl) phosphate; BMPP, bis(2-methylphenyl) phosphate; DBUP/DIBP, composite of dibutyl phosphate and di-isobutyl phosphate; DPHP, di(2-propylheptyl) phthalate; DPRP, dipropyl phosphate; ECHO, Environmental influences on Child Health Outcomes; OPE, organophosphate ester flame retardant and plasticizer.

**Figure S6**. Coefficient plots of leave-one-out results for SRS total T-score (adjusted models)

Abbreviations: BBOEP, bis(butoxyethyl) phosphate; BCPP, bis(1-chloro-2-propyl) phosphate; BCETP, bis(2-chloroethyl) phosphate; BDCPP, bis(1,3-dichloro-2-propyl) phosphate; BEHP, bis(2-ethylhexyl) phosphate; BMPP, bis(2-methylphenyl) phosphate; CANDLE, Conditions Affecting Neurocognitive Development and Learning in Early Childhood; CI: confidence interval; CIOB, Chemicals in Our Bodies; DBUP/DIBP, composite of dibutyl phosphate and di-isobutyl phosphate; DPHP, di(2-propylheptyl) phthalate; DPRP, dipropyl phosphate; EARLI, Early Autism Risk Longitudinal Investigation; ECHO, Environmental influences on Child Health Outcomes; IKIDS, Illinois Kids Development Study; MADRES, Maternal and Developmental Risks from Environmental and Social Stressors; MARBLES, University of California – Markers of Autism Risk in Babies – Learning Early Signs; NHBCS, New Hampshire Birth Cohort Study; NYU CHES, New York University Children’s Health and Environment Study; OPE, organophosphate ester flame retardant and plasticizer; PETALS, Pregnancy Environment and Lifestyle Study; SRS, Social Responsiveness Scale; TIDES, The Infant Development and the Environment Study; VCSIP, Vitamin C to Decrease the Effects of Smoking in Pregnancy on Infant Lung Function.

**Figure S7.** Coefficient plots of leave-one-out results for Clinical Autism Spectrum Disorder (ASD) diagnosis (adjusted models)

Abbreviations: ASD, autism spectrum disorder; BBOEP, bis(butoxyethyl) phosphate; BCPP, bis(1-chloro-2-propyl) phosphate; BCETP, bis(2-chloroethyl) phosphate; BDCPP, bis(1,3-dichloro-2-propyl) phosphate; BEHP, bis(2-ethylhexyl) phosphate; BMPP, bis(2-methylphenyl) phosphate; CANDLE, Conditions Affecting Neurocognitive Development and Learning in Early Childhood; CI: confidence interval; CIOB, Chemicals in Our Bodies; DBUP/DIBP, composite of dibutyl phosphate and di-isobutyl phosphate; DPHP, di(2-propylheptyl) phthalate; DPRP, dipropyl phosphate; EARLI, Early Autism Risk Longitudinal Investigation; ECHO, Environmental influences on Child Health Outcomes; IKIDS, Illinois Kids Development Study; MADRES, Maternal and Developmental Risks from Environmental and Social Stressors; MARBLES, University of California – Markers of Autism Risk in Babies – Learning Early Signs; NHBCS, New Hampshire Birth Cohort Study; NYU CHES, New York University Children’s Health and Environment Study; OPE, organophosphate ester flame retardant and plasticizer; PETALS, Pregnancy Environment and Lifestyle Study; TIDES, The Infant Development and the Environment Study; VCSIP, Vitamin C to Decrease the Effects of Smoking in Pregnancy on Infant Lung Function.

**Figure S8**: Comparison of DPHP and BDCPP concentrations across studies

Note: The NHANES study did not report 25 percentile values of the distribution so only the 50-75^th^ percentiles are shown here. Abbreviations: BDCPP, bis(1,3-dichloro-2-propyl) phosphate; DPHP, di(2-propylheptyl) phthalate

**References**

1.	Harrod CS, Fingerlin TE, Chasan-Taber L, Reynolds RM, Glueck DH, Dabelea D. Exposure to prenatal smoking and early-life body composition: the healthy start study. *Obesity (Silver Spring)*. Jan 2015;23(1):234-41. doi:10.1002/oby.20924

2.	Zhu Y, Hedderson MM, Feng J, Mevi AA, Ferrara A. The Pregnancy Environment and Lifestyle Study (PETALS): a population-based longitudinal multi-racial birth cohort. *BMC Pregnancy Childbirth*. Apr 17 2017;17(1):122. doi:10.1186/s12884-017-1301-0

3.	Bastain TM, Chavez T, Habre R, et al. Study Design, Protocol and Profile of the Maternal And Developmental Risks from Environmental and Social Stressors (MADRES) Pregnancy Cohort: a Prospective Cohort Study in Predominantly Low-Income Hispanic Women in Urban Los Angeles. *BMC Pregnancy Childbirth*. May 30 2019;19(1):189. doi:10.1186/s12884-019-2330-7

4.	Gilbert-Diamond D, Emond JA, Baker ER, Korrick SA, Karagas MR. Relation between in Utero Arsenic Exposure and Birth Outcomes in a Cohort of Mothers and Their Newborns from New Hampshire. *Environ Health Perspect*. Aug 2016;124(8):1299-307. doi:10.1289/ehp.1510065

5.	LeWinn KZ, Bush NR, Batra A, Tylavsky F, Rehkopf D. Identification of Modifiable Social and Behavioral Factors Associated With Childhood Cognitive Performance. *JAMA Pediatrics*. 2020;174(11):1063-1072. doi:10.1001/jamapediatrics.2020.2904

6.	McEvoy CT, Milner KF, Scherman AJ, et al. Vitamin C to Decrease the Effects of Smoking in Pregnancy on Infant Lung Function (VCSIP): Rationale, design, and methods of a randomized, controlled trial of vitamin C supplementation in pregnancy for the primary prevention of effects of in utero tobacco smoke exposure on infant lung function and respiratory health. *Contemp Clin Trials*. Jul 2017;58:66-77. doi:10.1016/j.cct.2017.05.008

7.	Hertz-Picciotto I, Schmidt RJ, Walker CK, et al. A Prospective Study of Environmental Exposures and Early Biomarkers in Autism Spectrum Disorder: Design, Protocols, and Preliminary Data from the MARBLES Study. *Environ Health Perspect*. Nov 2018;126(11):117004. doi:10.1289/EHP535

8.	Newschaffer CJ, Croen LA, Fallin MD, et al. Infant siblings and the investigation of autism risk factors. *J Neurodev Disord*. Apr 18 2012;4(1):7. doi:10.1186/1866-1955-4-7

9.	O'Connor T, Best M, Brunner J, et al. Cohort profile: Understanding Pregnancy Signals and Infant Development (UPSIDE): a pregnancy cohort study on prenatal exposure mechanisms for child health. *BMJ Open*. Apr 1 2021;11(4):e044798. doi:10.1136/bmjopen-2020-044798

10.	Liu R, DeSerisy M, Fox NA, et al. Prenatal exposure to air pollution and maternal stress predict infant individual differences in reactivity and regulation and socioemotional development. *J Child Psychol Psychiatry*. Nov 2022;63(11):1359-1367. doi:10.1111/jcpp.13581

11.	Eick SM, Enright EA, Geiger SD, et al. Associations of Maternal Stress, Prenatal Exposure to Per- and Polyfluoroalkyl Substances (PFAS), and Demographic Risk Factors with Birth Outcomes and Offspring Neurodevelopment: An Overview of the ECHO.CA.IL Prospective Birth Cohorts. *Int J Environ Res Public Health*. Jan 16 2021;18(2)doi:10.3390/ijerph18020742

12.	Morello-Frosch R, Cushing LJ, Jesdale BM, et al. Environmental Chemicals in an Urban Population of Pregnant Women and Their Newborns from San Francisco. *Environ Sci Technol*. Nov 15 2016;50(22):12464-12472. doi:10.1021/acs.est.6b03492

13.	Trasande L, Ghassabian A, Kahn LG, et al. The NYU Children's Health and Environment Study. *Eur J Epidemiol*. Mar 2020;35(3):305-320. doi:10.1007/s10654-020-00623-6

14.	Barrett ES, Sathyanarayana S, Janssen S, et al. Environmental health attitudes and behaviors: findings from a large pregnancy cohort study. *Eur J Obstet Gynecol Reprod Biol*. May 2014;176:119-25. doi:10.1016/j.ejogrb.2014.02.029
